# Effects of Topper Training on psychosocial problems, self-esteem, and peer victimisation in Dutch children: A randomised trial

**DOI:** 10.1371/journal.pone.0225504

**Published:** 2019-11-27

**Authors:** Lilian Vliek, Geertjan Overbeek, Bram Orobio de Castro

**Affiliations:** 1 Knowledge Centre of Topper Training Foundation, Almere, The Netherlands; 2 Research Priority Area Yield, Research Institute of Child Development and Education, University of Amsterdam, Amsterdam, The Netherlands; 3 Department of Developmental Psychology, Utrecht University, Utrecht, The Netherlands; University of La Rioja, SPAIN

## Abstract

Most interventions aimed at improving social interactions either target internalising or externalising problem behaviour in children. However, a recent review shows that a transdiagnostic approach might fit better to the diversity of problems within a group and within an individual (comorbidity). We examined the effectiveness of a transdiagnostic intervention, called Topper Training: a cognitive behavioural intervention in the peer group with parents included, that targets both internalising and externalising behaviour problems. A randomised trial with a waiting list control group was conducted, using 132 children with mild to severe psychosocial problems. Children were randomised into 77 intervention and 55 waiting list children (50% boys; age = 8–11 years). GLM repeated measures analyses yielded significant intervention effects directly after the training on parent-reported (but not teacher-reported) emotional symptoms (Cohen’s *d* = .70), peer relationship problems (*d* = .41), and impact of these problems (*d* = .59). Significant effects were also found for child-perceived peer victimisation (*d* = .62), self-esteem (*d* = .45) and teacher-reported conduct problems (*d* = .42). Parent-reported effects on emotional, conduct problems and impact of the problems and child-reported effects on self-esteem were clinically relevant. No significant effects of Topper Training were found for prosocial behaviour and bullying. Within-participant t-tests in the intervention group between post-intervention and follow-up indicated that effects extended over a six-month follow-up period. Depression decreased significantly from post-test to follow-up. In conclusion, children with mild to severe internalising and/or externalising problems can benefit from the transdiagnostic Topper Training intervention.

## Introduction

Children spend a lot of time interacting with other children and this is not an easy job for all of them. Children can show aggressive reactions but also depressive and withdrawn reactions to daily life challenges such as trying to belong to a group, bullying, denial or other social situations [1]. To a certain level, these challenges belong to normal development. From a biopsychosocial perspective [2], the interaction of biological, psychological and social aspects can create vulnerability for children in certain environments to develop problems. When these interaction processes continue, the first symptoms or psychosocial problems can develop. With psychosocial problems we mean emotional (or internalising), conduct (or externalising) and social peer problems, following the definition of Theunissen [3]. The prevalence of psychosocial problems in Dutch 8- to 12- year old children is 10% [4]. More specific: 8% of Dutch primary school children shows conduct problems and 12% shows emotional problems (parent report) [5]. Early conduct and emotional problems are found to be important predictors of depression, delinquency, school dropout and psychological disorders later on in life [6]. Reducing these problems at an early age with indicative preventive interventions directed at psychosocial problems may prevent escalation into severe problems that are harder to treat [7] and save society from the associated costs and risks [8].

Many of the interventions directed at social interactions target one kind of problem behaviour: either internalising *or* externalising problems. In a recent review, Marchette and Weisz [9] argue that there is a mismatch between this focal treatment on single problems and treatment of children in real-world clinical care. Children very frequently have comorbid problems and are thus diagnostically heterogeneous. This notion is in line with findings of Caspi et al. [10]. Their study indicated that psychiatric disorders could best be explained using one general psychopathology factor: the p factor. They argued that this p factor makes it difficult to find strongly effective treatments for individual mental disorders. Thus, working with transdiagnostic approaches may be a better idea. With a transdiagnostic approach we mean an intervention in which a guiding therapeutic strategy is universally applied across the range of presenting conditions (see also [11]). This approach has some advantages above single-diagnosis protocols. First, the single-diagnosis protocols do not provide guidance on how to address co-occurring diagnoses (e.g. [12]. Some studies have indeed shown that these protocols demonstrate poorer outcomes for the primary disorder for the individuals presenting with more than one diagnosis (e.g. [13]). Another advantage is that therapists need only to receive training in one protocol rather than costly and time-intensive training for multiple interventions [14].

In delineating a potentially effective transdiagnostic approach, it is important to delineate key intervention strategies and focus points of the intervention. Earlier studies have identified specific effective intervention strategies that seem to work in decreasing internalising and externalising problems in youth. Cognitive behavioural interventions [15, 16], parent-child training [17] and peer group interventions [18] are generally found to be effective ways of stimulating social interactions. Social competence calls upon a complex set of skills and competencies. Therefore, in the Handbook of Youth Prevention Science [19], we recommended to focus on several factors to include in preventive interventions for psychosocial problems. In sum, these are relational factors (such as involving peers, diminish reinforcement of negative behaviour, give dominant children insight into their actual popularity, train parents and teachers) and child factors (such as practice social skills, train social information processing, emotion regulation, and realistic self-esteem. In addition to focussing on these risk and protective factors, we recommended to focus on the authentic desire of children (their positive intentions) and on their feeling of responsibility for their behaviour (at a developmentally appropriate level). These last focus points are similar to the concept of autonomy in the Self-Determination Theory [20, 21]. The theory is based on the assumption that people have natural tendencies. They want to grow, to master challenges and to integrate new experiences into a coherent sense of self. This does not occur automatically. When people feel satisfied in their basic psychological needs: autonomy, competence and relatedness, they will develop and function effectively and experience wellness. Whether or not these needs are satisfied is depending on the child’s interaction with the environment. Following this theory, internalising and externalising behaviour can be understood in terms of reactions to basic needs being thwarted [20]. This implies that in trying to decrease internalising or externalising behaviour and to increase socially competent behaviour, an intervention should stimulate not only the above-mentioned developmental child and relational factors, but also children’s feelings of autonomy, competence and relatedness.

### Topper training

This study examines the effectiveness of Topper Training (“Kanjertraining” in Dutch; [22]) in a mental health care setting. Topper Training is a well-known intervention in the Netherlands [23]. This training is directed at children with internalising as well as externalising behaviour and takes into account the importance of motivation, autonomy, competence and relatedness. It includes cognitive behavioural techniques and is directed at the child and its environment: classmates, school and parents. More specifically, the training is given in three settings: 1) as a universal intervention in primary and secondary schools by teachers; 2) as a curative classroom intervention in disrupted classes by psychologists; and 3) as an indicative preventive intervention in mental health care centres to children and their parents when there is a concern about the social development of the child because of mild to severe psychosocial problems. In all three settings, the intervention is transdiagnostic: targeting mild to severe internalising and externalising behaviour (problems). The program focuses on the attitudes and behaviour of children and parents and in the school settings of educators and the head of the school. Variants of the Topper Training method to create positive group climates are also widely used in sports associations, out-of-school childcare, churches and entire neighbourhoods. In this article, the intervention is studied in the context of a mental health care centre, as an indicated preventive intervention.

The training takes into account the importance of motivation and autonomy by reminding children of their positive intentions and motivation to behave prosocially and by making children aware of their ability to choose their own (autonomous) behaviour. The main method that is used to foster these skills is the use of four caps.

The white cap stands for authentic behaviour on a base of trust in oneself and in the other. Different coloured caps in combination with the white cap cover many ways in which people feel authentic and act based on trust. The black cap in combination with the white cap represents power, leadership, initiative taking and spirit. In the same way, the yellow with white cap represents modesty and being sensitive to others needs and feelings. The combination of red and white cap represents humour (with respect for all parties) and being able to relativise.

All coloured caps have their pitfall. Problematic behaviour (internalising and externalising behaviour) is seen as non-authentic behaviour because, most of the time, it is not the desire of the child to behave without the white cap [22]. The black cap without the white cap stands for aggressive and dominating behaviour; the yellow cap stands for shy, anxious and depressed behaviour; the red cap stands for annoyingly funny, careless and 'accomplice-like' behaviour. A key point is that while children may behave in accordance with the role that belongs to a certain cap, they are not identified or labelled as such. In other words: the cap refers to behaviour, not to a personal trait. Difficult social situations are acted out in role-plays, for example bullying situations that have arisen in the class. The caps can also be used outside the training sessions: children, teachers and parents can ask children “Which cap are you wearing?” so as to make children more conscious of their behaviour. Subsequently, they can ask the child whether he/she would like to put on the white cap. A more detailed description of the theoretical ground and method of Topper Training can be found in [23]. The concept of the white cap is comparable to the view of Self-Determination Theory (SDT) where people have a natural tendency to express and develop themselves. The basic needs in SDT can be linked to the method and theoretical grounds of Topper Training: autonomy (Topper Training says “be yourself, make your own choices”), feeling of competence (by practicing social skills and increasing the feeling of control over one’s life) and relatedness (exercises in interaction with others and trust in others).

### Previous research on Topper training

In a quasi-experimental study, the effectiveness of Topper Training was established in a classroom context [23]. Classes (third to sixth grade; age range 8 to 13 years) designated as problematic by their teacher and/or the head of the school were trained by a psychologist. Parents and heads of schools were actively involved and the teachers were coached. The intervention consisted of an average of 15 training hours. Fourteen trained classes (n = 353) were compared to fourteen control classes (n = 343) from the same primary schools. Multilevel analyses revealed medium to large effects on classroom climate: relationship with the teacher, perceived social acceptance by classmates and disruptive behaviour according to the teacher. Cohen’s effect sizes ranged from .66 to 1.55. At the individual level, trained children showed improvements in self-reported prosocial behaviour, depressed mood and self-esteem when compared to the control children. Effect sizes ranged from .20 to .41.

In another quasi-experimental study in a mental healthcare setting [24], 185 trained children were compared to 39 waiting list control children (all between 8 and 11 years old). The training was directed at children with mild to severe psychosocial problems and their parents. After ten 90-minute sessions, the children showed significant decreases in parent-reported internalising and externalising problems, aggression, withdrawn-depressed behaviour, social problems and their problems in general. Marginally significant effects were found for attention problems, anxious-depressed problems and somatic problems. Effect sizes ranged from .26 to .46.

These studies were done under real-world conditions: participants applied for the training as usual and the training was given as usual. An advantage of this approach is that the results are easily transferrable to daily practice. This is crucial because Topper Training is already widely implemented in the Netherlands.

### The present study

The quasi-experimental design of both earlier studies did not allow strong conclusions to be drawn on the causal effect of the intervention. To overcome this limitation, the aim of this study was to examine Topper Training effects with a more stringent test: a randomised trial. Earlier studies were based on child, parent or teacher reports. The present study uses multiple informants in one study: parents, teachers and children. Moreover, we added a follow-up measurement after six months. The main question is: Is Topper Training effective for 8- to 11 year olds with mild to severe problems in social interaction in a mental health care setting, and does this effect remain for half a year?

We conducted the research in a mental healthcare centre in Almere, a medium-sized city in a central region of the Netherlands. The target population in this mental healthcare centre consisted of children with mild to severe problems in social interaction. Our primary hypothesis was: Topper Training can effectively reduce emotional problems and, conduct problems. Moreover, we expected that Topper Training could increase self-worth and prosocial behaviour and could decrease peer problems, depression, bullying and victimisation and could help children to cope more adequately with the challenges or problems they faced. Therefore, we hypothesised that Topper Training would also reduce the impact that problems have on the lives of children. Moreover, we hypothesised that the effects would sustain until 6-months follow-up.

## Materials and methods

### Design

We used a randomised trial with two conditions (intervention group and waiting list control group), three measurement points (pre, post and six-month follow-up) and three informants (child, teacher, parents). Individual children were randomly assigned to the intervention group or to the waiting list group in a 3:2 ratio using a simple randomisation procedure (a throw of the dice, 6 was ‘throw again’). The 3:2 allocation ratio was chosen for practical reasons: in September 2010 and 2011 three groups could start and in February 2011 and 2012 only two groups could start with the training (which was the delayed intervention of the waiting list group). To recruit sufficient numbers of participants, children were recruited in two time periods, between February 2010 and August 2010 and the same period one year later. The intervention started half yearly in September 2010, February 2011, September 2011 and February 2012 so that the waiting list group received the intervention six months after the intervention group.

All parents signed a consent form to indicate that they agreed to participate in the study. The study was approved by the Ethics review board of the Faculty of social and behavioural sciences of the University of Amsterdam, The Netherlands. The trial was registered under number 2014-CDE-3827 as “Effectiveness of Topper Training”. This trial is listed on the ISRCTN registry as “Effects of Topper Training on psychosocial problems, self-esteem, and peer victimisation” with study ID ISRCTN14967790, see http://www.isrctn.com/ISRCTN14967790. We registered the trial after participant recruitment, because the training was financed as preventive and not part of clinical mental health care at that time. There are no other clinical trials at the moment for this intervention.

### Participants

Children were recruited in primary schools and public health institutions in Almere in the Netherlands. Schools and institutions received posters and were informed about the possibility for children to participate in the Topper Training for free. The posters were directed at parents who were concerned about their child because of problems regarding social interaction. Examples of these problems were given, such as victimisation, low self-esteem, socially unskilful behaviour and aggressive behaviour.

**Fig 1:**
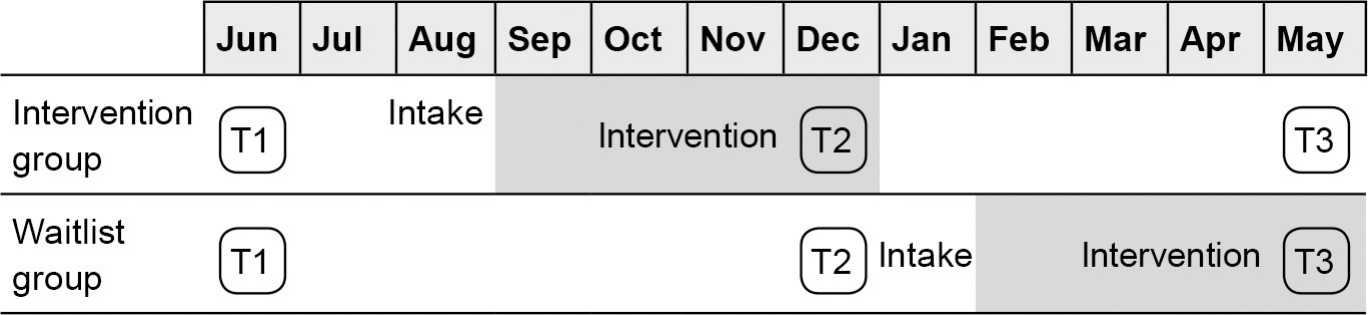
Participant flow chart. n = number of children, T1 = measurement 1, T2 = measurement 2, T3 = Measurement 3, CDI = Child Depression Inventory, CBSK = The Dutch version of the Self-Perception Profile for Children.

Eligible participants were children who were in primary school, were aged between 8 and 11 years, experienced internalising and/or externalising problems in social interactions and were motivated to follow the training programme (as were their parents). These criteria were exactly the same as those used in the daily practice of the training. A total of 140 families were eligible for inclusion in the study (see Fig 1). Of those, 134 families (96.3%) expressed their desire to participate in the study and gave their permission. The 134 children from these families were randomly assigned to the intervention group (n = 79) and waiting list group (n = 55). Two children from the intervention group did not report any problems at the interview stage and therefore chose not to participate in the intervention. At post-intervention (T2), all of the remaining children (77 intervention and 55 waiting list children) were still participating in the study. A sensitivity analysis in Gpower indicates that with power of .95, the sample size allowed for detection of modest effect sizes of *d* > .31 (effect size *f* > .157).

**Fig 2:**
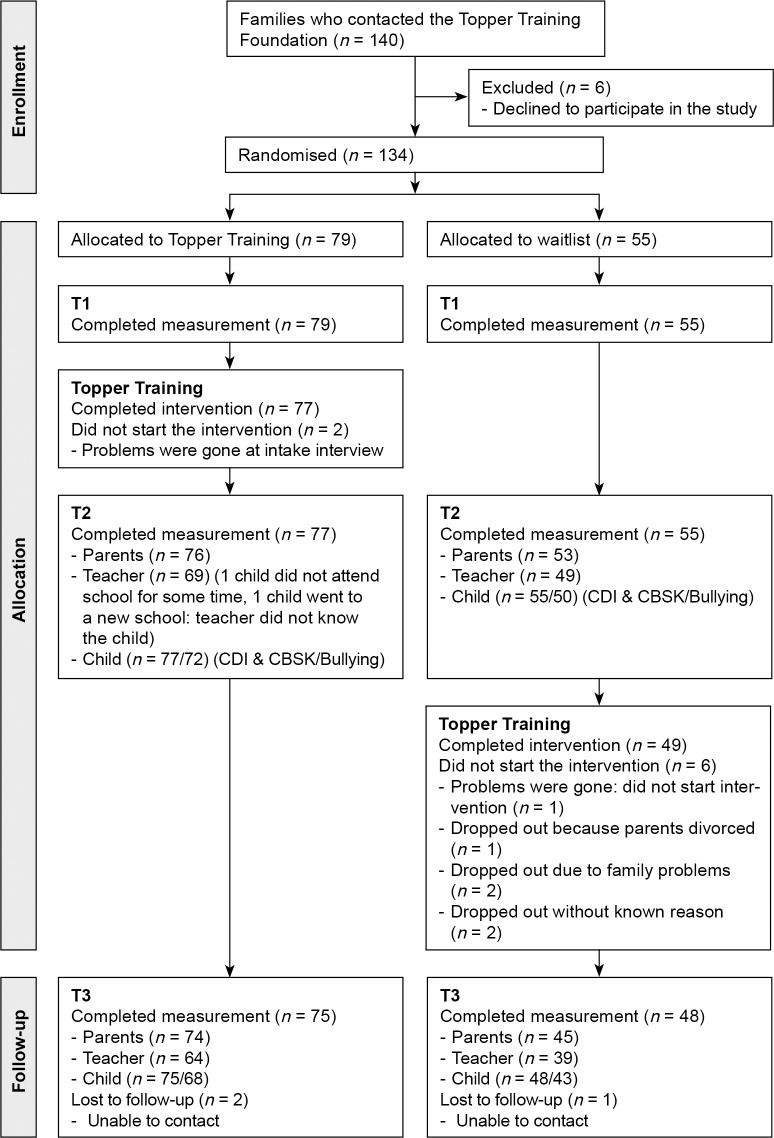
Measurement (T1, T2, T3) and intervention occasions starting in 2010 and 2011.

The waiting list group received the intervention half a year later than the intervention group, see Fig 2. By that time, one child had decided not to participate in the intervention because the previously reported problems were no longer apparent. Five other (waiting list) children dropped out during the intervention: one child dropped out because the parents were in the process of a divorce, two children dropped out because of family problems and two children dropped out of the intervention for other, unknown reasons. All of these six children were included in the second time point before their intervention and dropped out thereafter. At the third measurement point, we were unable to contact two other children in the intervention group and one in the waiting list group.

Baseline demographic and clinical characteristics of the intervention and waiting list groups are shown in Table 1. Clinical problems that were the most reported were emotional symptoms, self-perceived victimisation and impact of the problems. According to the parents, about 10% of the children were diagnosed as having ADHD or ADD, one child had an anxiety disorder, one child had a disorder in the autistic spectrum and one child had attachment problems. The children with ADHD or ADD were prescribed medication for their condition.

**Table 1:** Baseline demographic and clinical characteristics of intervention and waiting list group.

	Intervention (*n* = 77)	Waiting list (*n* = 55)
Age (years)	9.51 (1.2)	9.2 (1.1)
Sex (male)	41 (53%)	25 (45%)
Socioeconomic status (SES)		
Low	5 (6.5%)	5 (9%)
Middle	32 (41.5%)	23 (42%)
High	40 (52%)	27 (49%)
Ethnicity		
Dutch	62 (80%)	40 (73%)
Western migrant	2 (3%)	5 (9%)
Non-Western migrant	18 (23%)	15 (27%)
Diagnosis ADD/ADHD	8 (10.4%)	5 (9%)
Anxiety disorder	1 (1.3%)	0
Autism spectrum disorder	0	1 (1.8%)
Attachment problems	1 (1.3%)	0
Clinical score on SDQ (PR)		
Emotional Symptoms	32 (42%)	19 (34%)
Conduct Problems	14 (18%)	8 (15%)
Peer Problems	27 (35%)	9 (16%)
Prosocial Behaviour	10 (13%)	7 (13%)
Impact of Problems	43 (56%)	24 (44%)
Topper questionnaire		
Self-perceived victimisation	40 (52%)	17 (31%)
Self-reported bullying	6 (8%)	5 (9%)
Self-worth (SPPC)	16 (21%)	15 (22%)
Depression (CDI)	18 (23%|)	8 (15%)

*Note*. Data are means (*SD*) or numbers (%). PR = Parent Report

The intervention and control groups did not differ in age (t(130) = 1.540, p = .126) or gender (Chi ^2^(1) = .779, p = .377). Mean age was 9.38 years (*SD* = 1.2). The percentage of boys was 50%. Level of education of the parents did not differ between the groups: the distribution of families in low, middle and high educational segments was 7%, 42% and 51% respectively (*Chi* ^*2*^(2) = .338, *p* = .845). Also, ethnic composition did not differ between the groups (*Chi* ^*2*^(2) = 3.349, *p* = .187), 78% was Dutch, 5% Western migrant and 17% non-Western migrant.

### Attendance

Attendance was high for both groups. The mean attendance for the intervention group over ten group sessions was 9.4 sessions (*SD* = .7), with 55% of the children attending all ten sessions, 35% attending nine sessions and 10% attending eight or seven sessions. Mean attendance during the intervention period of the waiting list group was 9.5 sessions (*SD* = .8), with 64% of the children attending all ten sessions, 24% attending nine sessions and 12% attending eight or seven sessions. Five intervention children filled in the post-intervention measurement after nine training sessions instead of ten. This was done because these children would not be able to fill in the questionnaires directly after the last training session. To ensure a post-test measure for these children, we chose to let them fill it in directly after the ninth session.

### Procedure

After recruitment, the pre-test measurement took place (T1, around June 2010 and for the second group June 2011), followed by the randomisation procedure. The intervention group then started with the intervention, followed by a post-intervention measurement (T2, December of the same year) directly after the last training session. Half a year later the follow-up measurement (T3 in May) took place. We organised a meeting for each training group to fill in all the questionnaires again. The waiting list group had to wait half a year after the first measurement and then completed the second measurement at the same time point as the intervention group. Thereafter, the waiting list group received the intervention, followed by the post-intervention measurement (T3) directly after the last training session (see Fig 2). All children had an interview that was planned after their pre-test preceding the intervention: after T1 for the intervention group and after T2 for the waiting list group. In the original study protocol, (see S2 File and S3 File), we planned to have two pre-test measurement occasions: in May and August. However, we decided to omit the August measurement. The reason was that for some of the children theses time points were too close to each other (because they registered in June or July). This made the August measurement less functional.

In general, parents filled in questionnaires at home before the intervention and at the mental health care centre after the last session. Teachers received the questionnaires from the parents and sent them back. Children filled in the questionnaires under supervision at the mental health care centre. The control group filled in pretest questionnaires at school under supervision, because the intake was half a year later. Completion of the questionnaires took about 15–20 minutes.

To motivate parents to fill in the questionnaires at three separate points in time, the training was offered for free (upon the precondition that all measurement occasions were completed) and parents received a report with the results for their child. Children, parents and teachers were all knowledgeable as to who was in the intervention condition and who was not: it was not possible to blind participants, parents or teachers.

### Intervention

Topper Training was provided by two trained psychologists with 5 and 7 years experience each in giving this training. The intervention consisted of ten 90-minute group sessions given every two weeks. Training groups contained a maximum of 15 children with internalising and/or externalising problems. In S5 File we give a more detailed description of the intervention.

### Measures

#### Strengths and Difficulties Questionnaire (SDQ)

Parents and teachers reported children’s problem behaviour on the SDQ [25, 26] a 25-item measure of problem behaviour and prosocial behaviour. We used the Emotional Symptoms scale (5 items), Conduct Problems scale (5 items), Peer Problems scale (5 items) and Prosocial Behaviour scale (5 items). We did not use the Attention and Hyperactivity scale in this study, because this is not one of the goals of Topper Training. Items were rated on a scale ranging from 0 (not true) to 2 (certainly true). In our sample, Cronbach’s alpha ranged between .69 and .81 for teacher reports and between .50 (mother report on Emotional Symptoms scale) and .71 for parent reports. Concurrent validity in a Dutch sample was established [26].

We used the extended SDQ with an additional impact supplement. This supplement provides an impact score, which is the sum of the scores on the distress and social incapacity items. The Impact score is found to discriminate better between community and clinic samples than symptom scores [27]. Pre-test scores of mother and father were strongly correlated (*r* between .51 and .79). We decided to take the average parent score by computing the mean score of father and mother. When the score of only one parent was available at a given point in time, we also used the score of that parent at the other time points for that child to ensure correct within-subject comparisons. This was the case for five training children and two control children.

#### Child Depression Inventory (CDI)

We assessed depressive symptoms through a Dutch translation [28] of the Children’s Depression Inventory (CDI) [29]. In this translation, one item from the original CDI concerning suicidal ideation (“I want to kill myself”) was replaced by two less precarious questions: I (never/sometimes/-often) think “I wish I was dead” and I (always/sometimes not/do not) think that life is worth living. This resulted in a 28-item questionnaire. For each item, children selected one of three statements indicating how they had felt over the past 2 weeks. The CDI has strong predictive, convergent and construct validity (e.g., [30, 31]), and was shown to have adequate internal consistency and test-retest reliability in previous studies [29, 32]. Cronbach’s alpha in the current sample was .85. On the basis of cut-off scores suggested by Kovacs [29], scores below 13 were rated as normal and scores of 16 or higher were rated as clinically depressed.

#### Self-Perception Profile for Children (SPPC)

We used the self-esteem scale from the Dutch version [33] of the Self-Perception Profile for Children [34]. This scale consists of 6 items. Each item consists of two opposing descriptions, from which children have to choose one and then indicate whether this is somewhat true or totally true for them. Accordingly, each item is scored on a four-point scale, with a higher score reflecting a more positive view of oneself. The Dutch version was found to be reliable (Cronbach’s alpha = .74 and test-retest reliability after four weeks was .74 [33]. The construct and concurrent validity was established in a Dutch sample [35]. Internal consistency in the current sample was .88. Scores below the 10^th^ percentile were rated as clinically low and above the 20^th^ percentile as normal. This translates into different scores for boys and girls: girls scored clinical below 16, boys below 17. Scores of 18 or higher were rated as normal for boys and girls.

#### Topper questionnaire

We used the Topper questionnaire [36] to measure bullying and self-perceived peer victimisation. Bullying was measured by the question: ‘I bully at school’ and self-perceived peer victimisation was measured by two questions: ‘I am afraid of being bullied’ and ‘I get bullied’, comparable to The Revised Olweus Bully/Victim Questionnaire [37]. For each statement children chose “totally not true,” “not really true,” “a little true” or “totally true” using a four-point Likert scale. Correlations with the other dependent variables of this study were in the expected direction and gave support for the concurrent validity of these questions, see S1 Table.

This questionnaire was filled in at home since supervision was not necessary. All other child questionnaires were completed under the supervision of a test assistant. Clinical relevance was measured by categorising children as ‘bully’ or ‘non-bully’ and ‘victim’ or ‘non—victim’. Children with a score below 3 (“totally not true” and “not really true”) were rated as non-victim or non-bully; children with a score of 3 or higher (“a little true” or “totally true”) were rated as victim or bully. This classification is comparable to the criterion (i.e. more than once or twice) used by Farrington and Ttofi [38] in their meta-analysis. The other scales of the Topper questionnaire were filled in, but we decided not to use them in this study because we already measured these aspects with other measures (CDI and SDQ).

The dependent variables correlated with each other in the expected directions and strength. Pearson correlations varied between *r* = -.72 (*p* < .001) and *r* = .46 (*p* < .001). For a complete overview of all correlations, see S1 Table.

### Data analyses

To test the immediate effects of Topper Training, we used Repeated Measures ANOVA with group (intervention, waiting list) as between group factor and time (T1, T2) as within group factor. A significant group x time interaction effect indicated an intervention effect. Effect sizes (Cohen’s *d*) were calculated by subtracting the T1-T2 change in the waitlist group from the T1-T2 change in the intervention group. And diving this by the pooled standard deviation of the T1-T2 difference scores (see [39]). Based on these difference scores we computed the confidence interval for Cohen’s d. To determine the clinical relevance of these results, we computed the proportion of children in each group that moved from the clinical to the normal range, based on the normative data of the instruments.

We used the three time points for the waiting list group to test for additional evidence of an intervention effect. The slope between T2 and T3 (the intervention period) was compared with the slope between T1 and T2 (waiting list period). The significance of the difference was tested with a quadratic interaction effect in a repeated measures analysis, while only including the waiting list group. A significant interaction in combination with inspection of the graphs for the direction of the interaction was used as an additional test for the intervention effect. To examine the extent to which immediate post-test change was maintained at the six-month follow-up in the intervention group, Paired-Samples t-tests were used on immediate post-test and follow-up scores.

We checked assumptions before conducting the repeated measures ANOVA. Most variables had a normal distribution. Some did not have a normal distribution, as was expected (e.g. depression). With a sample size of more than 30, this does not give a bias in the analyses (central limit theorem). Sphericity is only applicable when comparing three time points; in this study we only compare two time points in the analyses. Scores are independent since the control and training groups did not have any contact and could not influence each other’s answers on the questionnaires.

## Results

### Baseline differences between intervention and waiting list groups

At baseline, the groups only differed on self-perceived peer victimisation (*t* (121) = 1.984, *p* = .05). The intervention group scored higher at baseline (*M* = 2.3, *SD* = 1.0) than the waiting list group (*M* = 2.0, *SD* = 0.9). We corrected for these pre-test differences by entering the pre-intervention score as a covariate in an ANCOVA on the intervention effect. Mean scores did not differ between the intervention and control group for any other variable, including bullying, depression, self-esteem or the parent and teacher SDQ scales (all *p*’s > .05).

### Immediate effects

**Fig 3:**
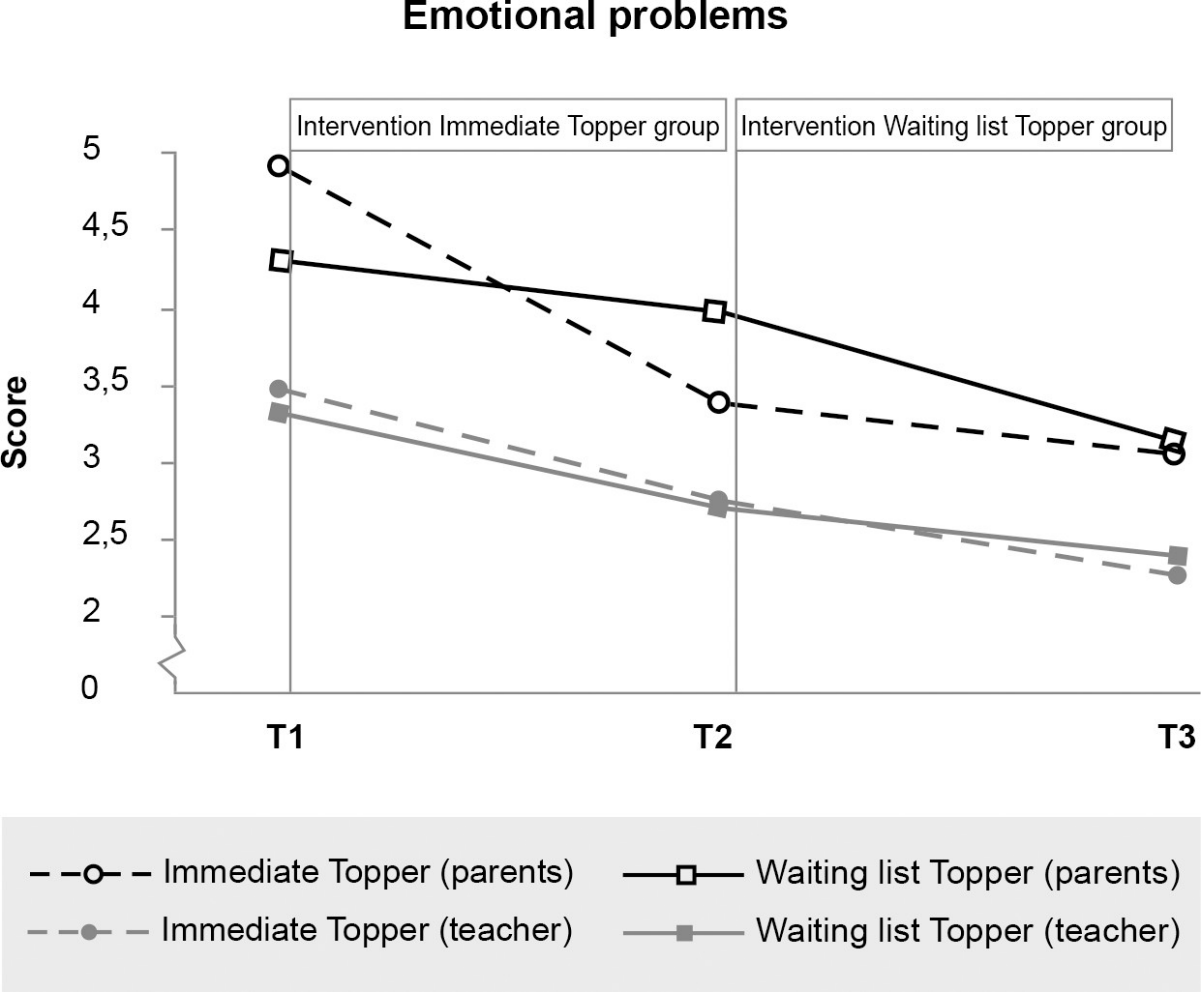
Significant effect of Topper Training on parent-reported (but not teacher-reported) emotional symptoms. N.B. The Figure plots a decrease of emotional symptoms during Topper Training period: between T1 and T2 for Immediate training group and between T2 and T3 for Waiting list group.

**Fig 4:**
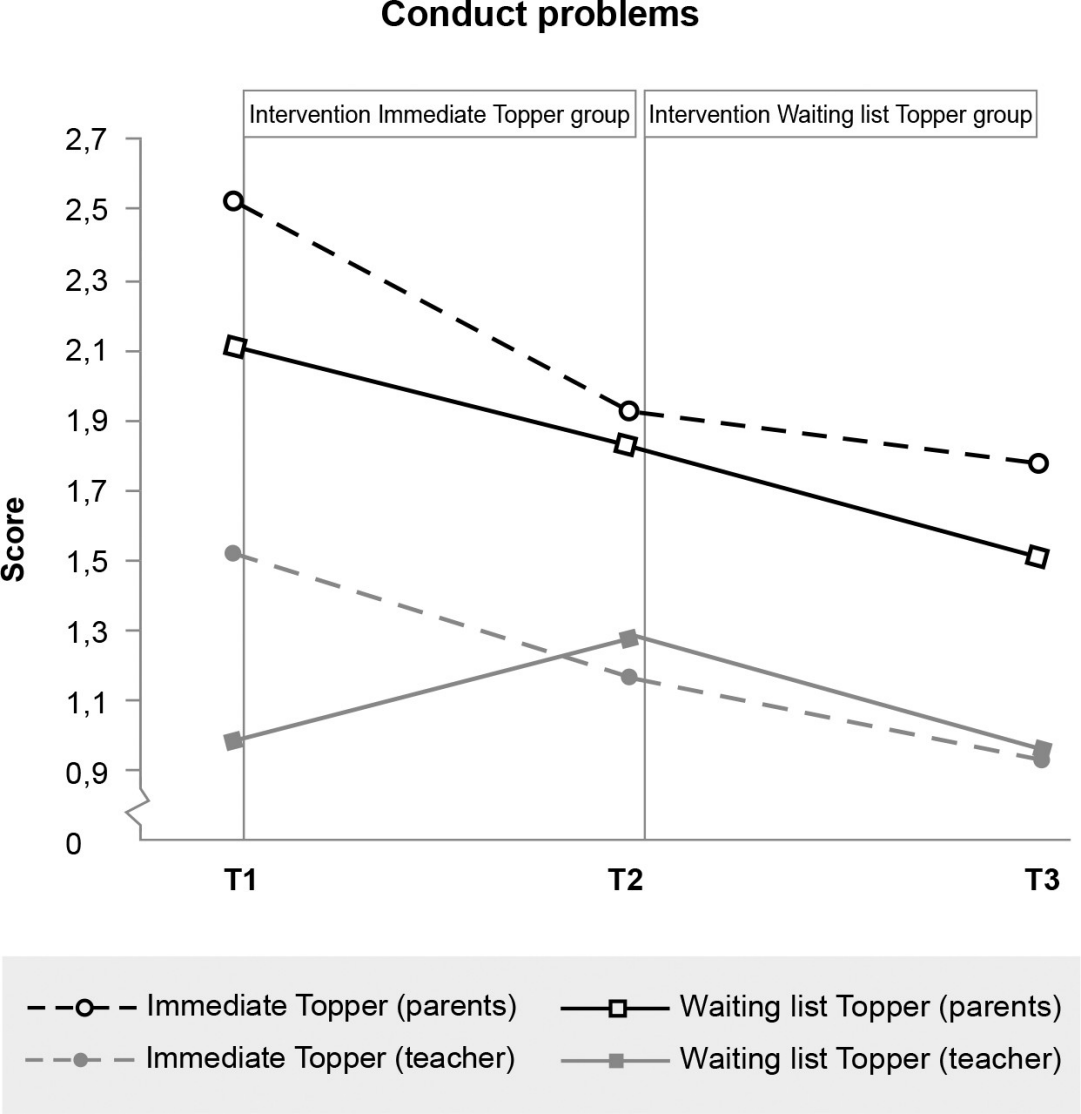
Significant effect of Topper Training on teacher-reported (but not parent-reported) conduct problems.

**Fig 5:**
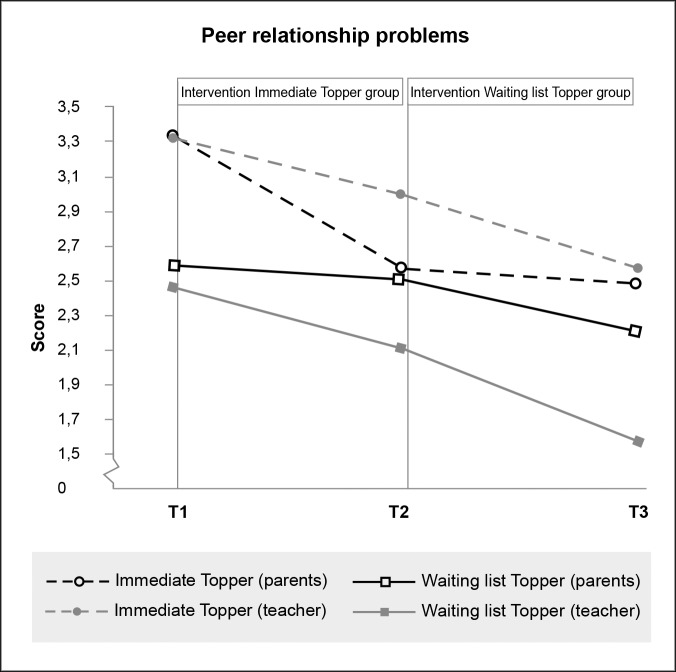
Significant effect of Topper Training on parent-reported (but not teacher-reported) peer relationship problems.

**Fig 6:**
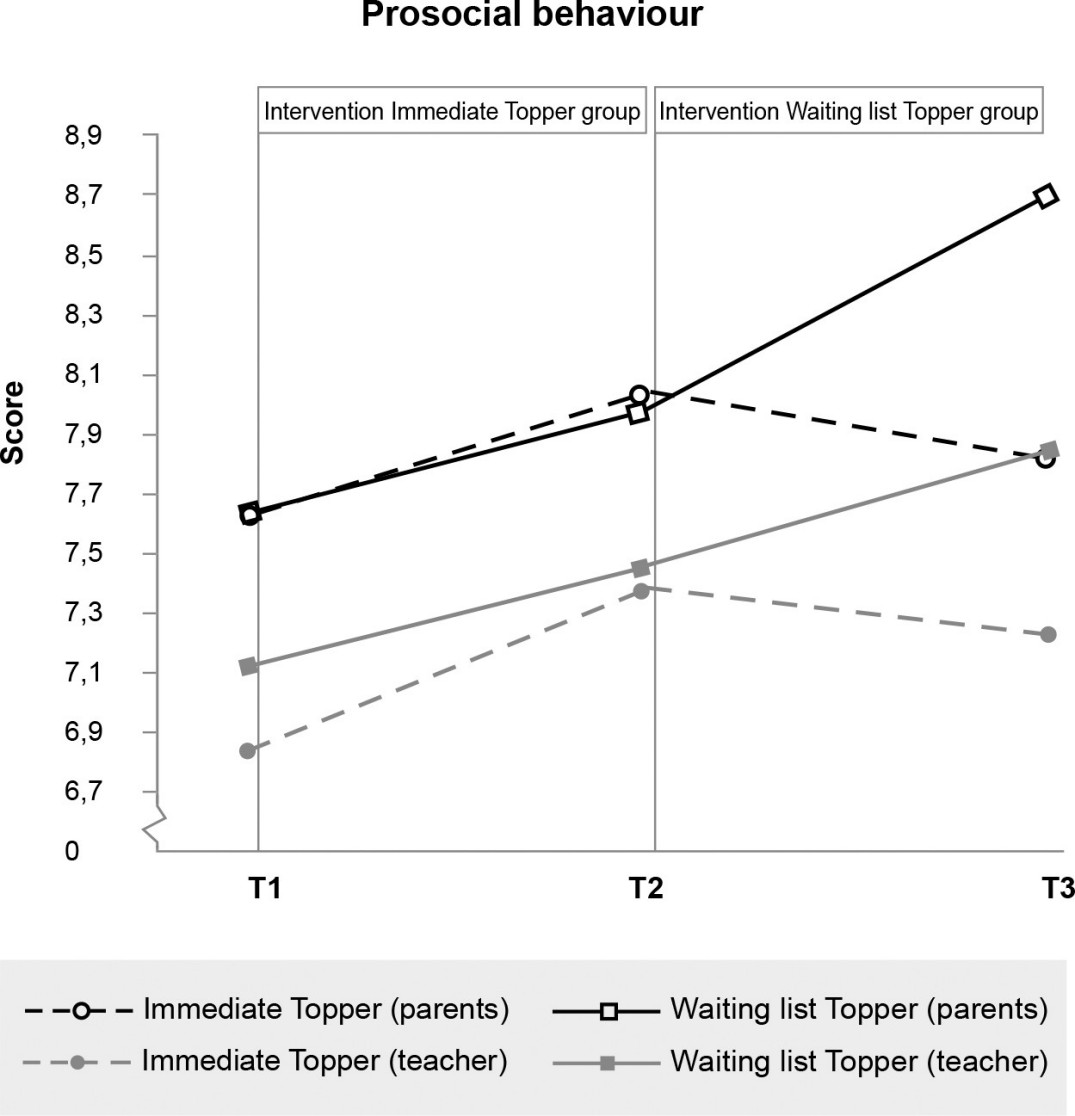
No significant effect of Topper Training on prosocial behaviour.

**Fig 7:**
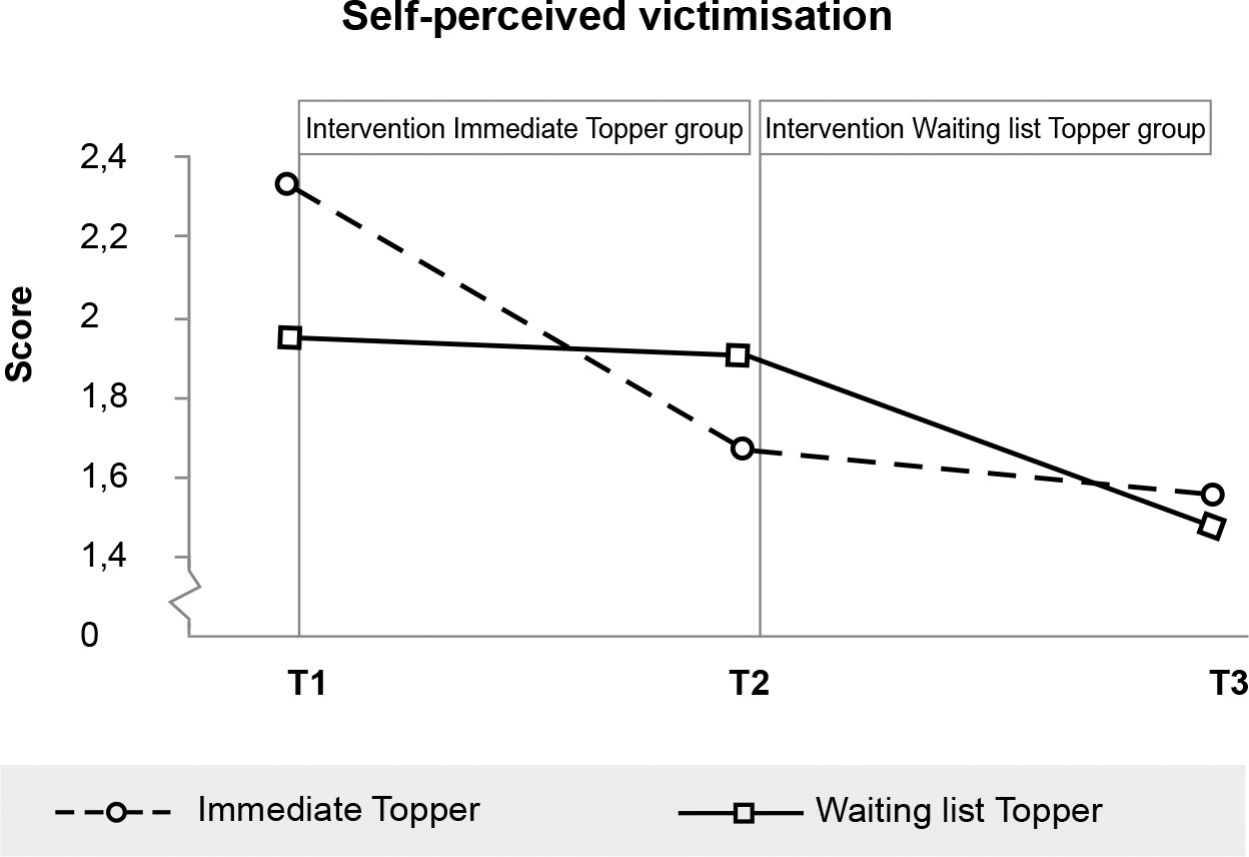
Significant effect of Topper Training on self-perceived peer victimisation.

**Fig 8:**
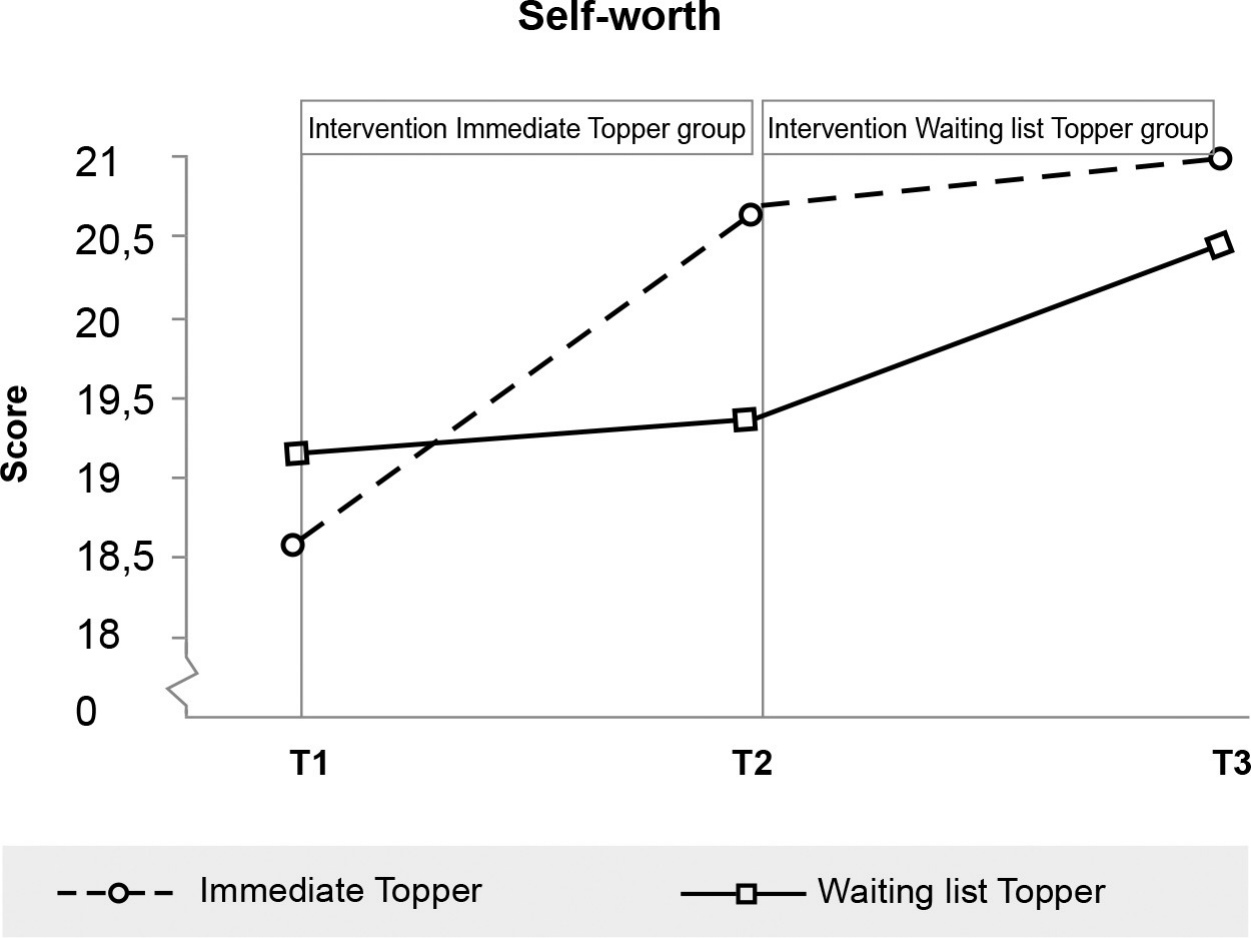
Significant effect of Topper Training on self-worth.

**Fig 9:**
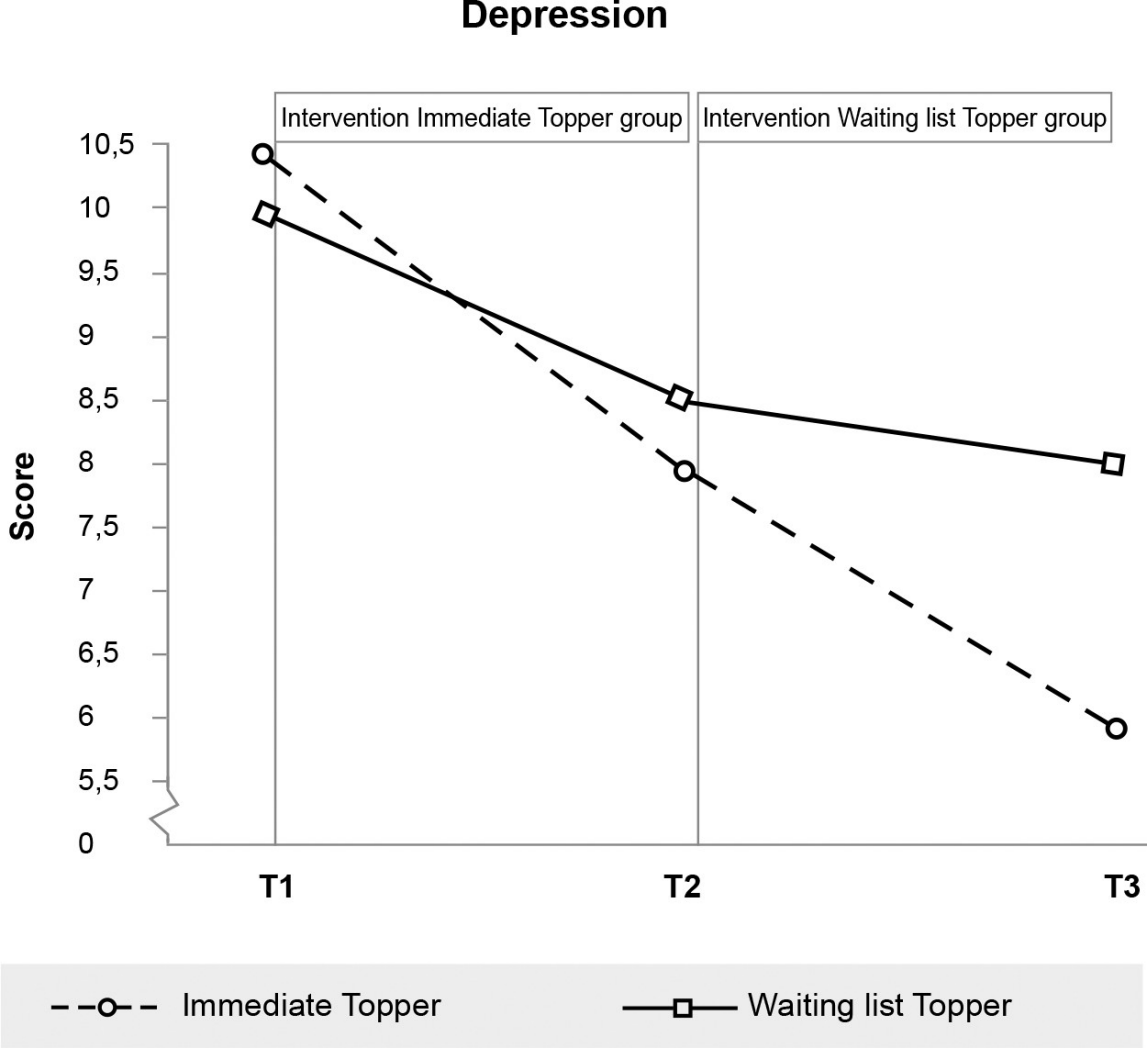
Significant effect of Topper Training on depression six months after the intervention. N.B. Depression significantly decreased between T2 and T3 for the Immediate Topper group. This could not be compared to a waiting list group, since the waiting list group received the training during this period.

Table 2 provides descriptive statistics for the intervention and waiting list groups at pre-intervention (T1), post-intervention (T2) and half a year later (T3). We plotted these mean scores in Figs 3–9 and in S1 and S2 Figs, calling the intervention group ‘Immediate Topper’ and the waiting list group ‘Waiting list Topper’. Table 3 provides the results from repeated measures analyses. The table shows Cohen’s *d* and its confidence intervals and the interactions between intervention group and time: the intervention effects. This interaction effect, indicating more positive change in the intervention group compared to the waiting list group, was significant for self-perceived peer victimisation *F*(1,119) = 6.66, *p* = .011, *d* = .62, self-worth *F*(1, 130) = 6.51, *p* = .012, *d* = .45, parent-reported (but not teacher-reported) emotional symptoms *F*(1,127) = 15.12, *p* = 1,62 * 10^−4^, *d* = .70, peer relationship problems *F*(1,127) = 5.14, *p* = .025, *d* = .41, and the impact of these problems *F*(1, 127) = 8.59, *p* = .004, *d* = .59) and teacher-reported (but not parent-reported) conduct problems *F*(1,118) = 4.95, *p* = .028, *d* = .42. No significant effects were found on self-reported bullying, depression or prosocial behaviour. No adverse effects were found: in all cases where no effects were found, the intervention group improved as much as the waiting list group did.

**Table 2:** Means and standard deviations (between brackets) for the intervention and waiting list group at T1, T2, and T3 (with clinical cut-offs of each subscale between brackets).

Subscale (clinical cut-off)		Intervention Mean (Standard deviation)			Waiting list Mean (Standard deviation)		
		T1	T2	T3	T1	T2	T3
Emotional symptoms (> 4) (Teach: > 5)	Parent	4.94 (2.1)	3.37 (2.0)	3.07 (2.2)	4.32 (2.2)	4.00 (2.1)	3.14 (1.8)
	Teach	3.52 (2.5)	2.72 (2.3)	2.33 (1.9)	3.35 (2.2)	2.76 (2.2)	2.41 (1.8)
Conduct problems (> 3)	Parent	2.54 (1.9)	1.93 (1.6)	1.83 (1.7)	2.13 (1.7)	1.83 (1.8)	1.53 (1.5)
	Teach	1.55 (2.0)	1.22 (1.5)	.97 (1.4)	1.00 (1.5)	1.30 (1.9)	1.00 (1.4)
Peer relationship problems (> 3) (Teach: > 4)	Parent	3.44 (2.4)	2.72 (2.2)	2.63 (2.1)	2.72 (2.0)	2.64 (1.8)	2.33 (1.5)
	Teach	3.41 (2.9)	3.13 (2.9)	2.72 (2.6)	2.59 (2.3)	2.22 (2.1)	1.69 (1.7)
Prosocial behaviour (< 5)	Parent	7.62 (1.9)	8.03 (1.8)	7.83 (2.1)	7.62 (1.73)	7.98 (1.5)	8.71 (1.4)
	Teach	6.87 (2.8)	7.38 (2.5)	7.25 (2.5)	7.16 (2.2)	7.45 (1.9)	7.87 (1.8)
Impact (> 1)	Parent	2.89 (2.0)	1.44 (1.9)	1.35 (1.8)	2.40 (1.6)	1.95 (1.5)	.81 (.96)
	Teach	2.1 (1.9)	1.28 (1.5)	1.16 (1.4)	1.63 (1.6)	1.06 (1.2)	.87 (1.1)
Self-perceived victimisation (> 2)	Child	2.34 (1.0)	1.68 (.8)	1.57 (.7)	1.96 (.9)	1.91 (1.0)	1.48 (.7)
Self-reported bullying (> 2)	Child	1.29 (.6)	1.31 (.7)	1.16 (.5)	1.32 (.8)	1.24 (.7)	1.09 (.3)
Self-worth (girl: < 16, boy: < 17)	Child	18.55 (4.6)	20.62 (3.6)	20.95 (4.1)	19.11 (5.7)	19.31 (4.2)	20.46 (3.6)
Depression (>15)	Child	10.40 (7.1)	7.96 (5.9)	5.95 (5.3)	10.02 (7.2)	8.60 (6.7)	7.98 (6.2)

*Notes*. Parent = parent report, Teach = teacher report, Child = child report

**Table 3:** Intervention effects: Time by condition interactions in repeated measures ANOVA’s.

		Parent report					Teacher report	
Results Intervention Effect	*F*	*p*	*d*	CI *Cohen’s d*	*F*	*p*	*d*	CI *Cohen’s d*
SDQ								
Emotional Symptoms	15.12	.000	.70	.33–1.06	.17	.684	.08	-0.29 - .44
Conduct Problems	1.47	.228	.20	-.15 - .55	4.95	.028	.42	.05 - .79
Peer Problems	5.14	.025	.41	.05 - .76	.04	.841	-.04	-.4 - .33
Prosocial Behaviour	.04	.835	.04	-.31 - .39	.26	.614	.10	-.27 - .46
Impact of Problems	8.59	.004	.59	.23 - .95	.68	.410	.16	-.21 - .52
	Child report				
Self-perceived victimisation	6.66	.011	.62	.25 - .99				
Self-reported bullying	4.18	.519	-.12	-.48 - .24				
Self-worth (SPPC)	6.51	.012	.45	.10 - .80				
Depression (CDI)	.97	.326	.17	-.18 - .51				

*Notes*. *d* = Cohen’s *d* effect size, CI = confidence interval. We corrected for pre-test differences in self-perceived victimisation by entering pre-test as a covariate in an ANCOVA on the post-intervention scores. Effect sizes (Cohen’s *d*) represent the T1-T2 change in Intervention group minus the T1-T2 change in the Waiting list group, divided by the pooled standard deviation for these difference scores.

*d* > 0 represents a positive effect of Topper Training.

### Additional within-group analyses of effects in the delayed intervention group

Additional analyses were conducted to test for the effects of Topper Training in the waiting list (i.e. delayed intervention) group. In these analyses changes in the outcome variables during the waiting list period were compared with changes in the same group during the intervention period using repeated measures ANOVA’s with the three time points and a quadratic contrast. Significant quadratic effects (indicating more improvement during the intervention period than during the waiting list period) were found for Self-perceived peer victimisation: *F*(1,42) = 4.23, *p* = .046, parent-reported Emotional symptoms: *F*(1,44) = 5.73, *p* = .021, and Impact of the problems: *F*(1,44) = 6.85, *p* = .011. No quadratic (intervention) effects were found for teachers: improvements experienced during the waiting list period were comparable with improvements during intervention, resulting only in significant linear effects.

### Maintenance of effects

Due to the delayed intervention design, no half-year follow-up data were available for the control group. We therefore tested whether outcomes were stable or improved from immediate post-intervention to the six-month follow-up for the intervention group separately. Within-participants t-tests indicated that scores did not change between immediate post-test and six-month follow-up for all scales on which immediate effects were found (see also Figs 3–8, S1 Fig and S2 Fig). While no immediate effect was found for depression (the waiting list group improved as much as the intervention group) the scores at follow-up were significantly lower than the scores at post-intervention, which may tentatively suggest that Topper Training reduces depression in the long run.

### Clinical relevance

Besides comparing average scores of the whole sample, it is interesting to test the effect of Topper Training specifically for children who scored in the clinical range. Clinical relevance of the results (i.e. the extent to which children scoring in the clinical range at pre-test showed movement to the normal range at post-test) is shown in Table 4. For most of the problem domains, the proportion of children scoring in the clinical range at baseline that moved to the normal distribution at post-test in the intervention group was substantial (30% to 70% across different measurements). Parent- and child-reported proportions of improvement were significantly higher in the intervention group than in the waiting list group for emotional and conduct problems, impact and self-esteem. Teacher-reported proportions were more similar in the intervention and waiting list groups, resulting in no statistical differences.

**Table 4:** Clinical relevance of results: Percentage of children who moved from clinical to normal range.

	Moved from clinical to normal range				Moved from clinical to normal range			
	Parent report				Teacher report			
	Intervention		Waiting list		Intervention		Waiting list	
	*n*	*%*	*n*	*%*	*n*	*%*	*n*	*%*
SDQ								
Emotional Symptoms	15 of 32	47*	3 of 19	16	7 of 10	70	3 of 5	60
Conduct Problems	9 of 14	64*	1 of 8	13	5 of 10	50	0 of 1	0
Peer Problems	9 of 27	33	2 of 9	22	7 of 18	39	4 of 7	57
Prosocial Behaviour	3 of 10	30	3 of 7	43	6 of 20	30	8 of 13	62
Impact of Problems	20 of 43	47*	3 of 24	13	9 of 26	35	9 of 16	56
	Child report							
Self- perceived victimisation	25 of 40	63	5 of 17	29				
Self-reported bullying	4 of 6	67	4 of 5	80				
Self-worth (SPPC)	11 of 16	69*	2 of 12	17				
Depression (CDI)	10 of 18	56	4 of 8	50				

*Notes*. *n* = number of clinical children that moved to normal range from pre-test to post-test

*p < .05 (of *Z*- statistic for difference of proportion of moved children between intervention and waiting list group).

## Discussion

In this study we evaluated the effects of the indicated preventive transdiagnostical intervention Topper Training in 8- to 11 year olds with mild to severe problems in social interaction in a mental health care setting. We hypothesised main effects on conduct as well as emotional problems. In line with this hypothesis, we found significant effects on parent reported emotional problems and on teacher reported conduct problems. We discuss possible explanations for the difference between parental and teacher report later on. Is it recommendable to give Topper Training to children with clinical emotional or conduct problems? Although only a subsample of the children could be included for this analysis, we found significant effects in our ‘clinical relevance analysis’. Almost half of the parents (47%) of children with clinical emotional problems reported that those problems were reduced to a normal range after the training, compared to 16% in the waiting list group. This is in line with our previous study in a mental health care setting [24], were parents reported a large effect for children with clinical internalising problems (*d* = .87).

Although parents did not report an effect on conduct problems in the whole sample, we found that 64% of the children with clinical conduct problems scored in the normal range at posttest (13% in waiting list group). This is in line with our previous study [24] that shows significant effects on parental reported clinical externalising problems and aggression. The overall effect on teacher reported conduct problems was in line with effects of Topper Training in a classroom context [23], where the teacher reported a large effect on disruptive behaviour at the classroom level.

Regarding the secondary outcomes, parents perceived a decline in peer problems (in line with our earlier findings [24]) and impact of those problems on the lives of the children, but no decrease in prosocial behaviour (in contrary to our previous study in a classroom context [23]). Additionally, half of the parents reported that the impact of the problems reduced from very high to normal (compared to 13%). Children experienced significant less peer victimisation and an increased self-worth after training (in line with effects in a classroom context [23], but no decrease on bullying (we did not measure this before). The training created a healthy self-worth for 69% of the children with clinical low self-worth (compared to 17%). All benefits of the training sustained up to half a year.

The effects of Topper Training on depression need a closer look. It appears that in the waiting list condition, children become less depressed over time without intervention. During the intervention, Topper Training does not seem to have an additional effect on depression. However, six months after the training, depression had declined even more in the training group. This suggests a sleeper effect. In the current design, we cannot make causal inferences on this, but it might indicate that Topper Training gives children tools to deal in a different way with social situations and that this gives them control over their lives and hence on the long run reduces depressed feelings. This would be in line with the mentioned ideas of Self-Determination Theory: stimulating autonomy, competence and relatedness (which Topper Training does) will contribute to well-being and hence reduce depressed mood.

Taken together, the results provide additional support for the effectiveness of Topper Training in 8- to 11-year-old children with mild to severe psychosocial problems, under real-world conditions. The effects are substantial and are in line with previous research on Topper Training in a mental healthcare setting and in a classroom setting [23, 24]. The discrepancies between parent- and teacher-reported effects are salient in this study. A surprising finding was that parents in the current study did not report significant improvements in their child’s conduct problems, while this *was* reported by the teachers, and while parent-reported conduct problems of the child *were* found to decrease in our earlier studies in a mental healthcare setting [24] and in a classroom context [23]. When we take a closer look at the data, it appears that the children in our sample had on average low conduct problems, showing a bottom-effect. Only about 15–18% of the children showed clinical conduct problems at pre-test according to parents. Topper Training was clearly effective for those children: about two-thirds of the children with clinical-level conduct problems at pre-test moved to the normal range at post-test (compared to 13% in the waiting list group). The fact that the improvement in conduct problems was clinically relevant but not statistically significant may be a consequence of the heterogeneity of the sample. To test whether Topper Training is effective for children with behavioural problems, a fully clinical sample would perhaps be more suited.

In contrast to parents, teachers did not seem to experience any effect of Topper Training on emotional symptoms, peer relationship problems and impact. Inspection of the data reveals that teachers experienced improvements in emotional and peer problems in control group children while they were waiting for the intervention. This might indicate that teachers may have been especially attentive to the children who were placed on a waiting list. This extra attention might have had a positive influence on the children, in that they may have felt more noticed and understood by the teacher, which in itself can lead to a reduction in emotional symptoms. Another explanation for the discrepancy between parent- and teacher-reports could be that teachers may be more sensitive to perceiving (changes in) conduct problems in a classroom context than to changes in emotional problems that are not readily observable. Yet another explanation might be that the new child social-emotional skills that have an effect on emotional symptoms and peer interaction are only practiced in the home context and have not yet been generalised into the school setting. This may take more time. Decreases in peer problems perceived by the teacher give support for this notion: six-month follow up scores of the teachers were comparable to post-test scores of the parents.

At first sight, another surprising finding was that Topper Training effectively reduced peer victimisation while the program did not affect levels of self-reported bullying. Perhaps this pattern of findings can be explained by the fact that there was very little self-reported bullying among the children in the current sample at baseline, so improvements could hardly be made. Future studies, with other criteria for inclusion, may test whether Topper Training reduces bullying by children who are selected for bullying behaviour. In addition, contrary to expectations, we did not find any significant effects on prosocial behaviour. Topper Training seems to have more effect on reducing problems than it does on stimulating positive behaviour. An explanation may be that children in this sample scored in the normal range at pretest on prosocial behaviour, on average (which in the SDQ means: being helpful and kind, sharing), which may have resulted in a ceiling effect.

For comparability with other studies, we used a significance level of *p* < .05, to identify results as either ‘significant’ or not. Our ‘significant’ *p*-values range from .011 to .046. Taking into account the applied nature of our study, these are fruitful results. However, if we translate those *p*-values to Bayes Factors (*B*), representing the amount of evidence supporting alternative hypothesis against the null hypothesis (see [40]), we see that most of our data cannot be considered “strong” evidence (Johnson [41] suggests to test at *p* < .005 (corresponding to a strong evidence criterion, or Bayes Factor of *B* > 14). Following that criterion, the effects on parent-reported emotional problems (*p* = 1,62 * 10^−4^ which corresponds to *B* = 260) and impact of the problems (*p* = .004: *B* = 17) give strong evidence for the difference in development between trained children and waitlist children. Thus, with this more stringent criterium we conclude that it is very likely that children receiving Topper Training show reductions in emotional problems and impact of the problems according to parents.

### Limitations and strengths

The present study provides a stringent test of the effectiveness of Topper Training, but it is still characterised by some limitations. One limitation of this study is that while we used multi-informant assessments, none of the informants were blind to condition. This might have influenced their responses. Another limitation is that the follow-up data for children who received the intervention directly could not be compared to a control group that did not undergo an intervention. A third limitation is that only a subset of our sample scored in the clinical range at pre-test: this made the sample size for calculating clinical relevance relatively small. While the clinical relevance of the current results is certainly promising, we need to test the effectiveness in a more clinical sample to generalise the results to a more clinical population. A fourth limitation of this study is that although we were generally able to use reliable and valid measures, bullying and peer victimisation were measured by only one and two questions, respectively. However, the Olweus Bully/Victim questionnaire [37] is used in many studies, and it too relies on two main questions (comparable to the ones we used in our study). Finally, to make our results more comparable to those obtained when using Olweus’ complete questionnaire, it would have been better if we had used similar response options to those used in previous studies, such as ‘not at all’, ‘only once or twice’, ‘two or three times a month’, ‘about once a week’, and ‘several times a week’. We did not do this because the questionnaire being used was part of the normal intervention intake procedure, with standard answering categories for all questions.

An important strength of this study is the random assignment of the children to either the intervention or waiting list group, which makes causal inference strong. In addition, the training was given under real-world conditions with routine provision of a training that is already widely implemented in this way. This makes the results significant in practical terms: this intervention in other mental healthcare centres by trained psychologists is likely to be effective. Results of an earlier study in these centres were found to be in line with the present findings [24]. Another strength of the study is the heterogeneity of the sample. This intervention is not only directed at and effective for children with either internalising or externalising problems, but rather is directed at the whole spectrum of psychosocial problems, taking into account one underlying general psychopathology factor: the p factor, as suggested by Caspi et al. [10].

### Conclusion and future research

Overall, these findings indicate that cognitive behavioural techniques taught in a peer group with an additional parent training and a focus on autonomy, competence and relatedness can be effective for children aged 8 to 11 years with psychosocial problems. Since Topper Training is widely implemented in the Netherlands and this study was done under real-world conditions, these results are promising in terms of the daily practice of this intervention for children with psychosocial problems. These effects were measured after 10 sessions, taking about five months in total. The intervention does not demand costly diagnostic tests, but can be followed without referral. This makes the intervention feasible.

As an additional step towards examining the effective elements of interventions for children with psychosocial problems, future research might examine the effectiveness of separate elements of the training. Moreover, a larger sample would enable us to examine the effectiveness of Topper Training in subsamples based on gender, age and severity of problems which would yield more information on the question for whom the intervention is more (or less) effective.

## Supporting information

S1 Fig Significant effect of Topper Training on parent-reported (but not teacher-reported) impact of the problems. (TIF)

S2 Fig No significant effect on self-reported bullying. (TIF)

S1 File Abstract in Dutch. (DOC)

S2 File Trial study protocol in Dutch. (DOC)

S3 File Trial study protocol in English. (DOC)

S4 File CONSORT checklist. (DOC)

S5 File Description of the intervention Topper Training. (DOC)

S1 Table Correlations of all outcome measures. (DOC)

## References

[1] Crick NR, Dodge KA. A review and reformulation of social information-processing mechanisms in children’s social adjustment. Psychological Bulletin. 1994;115(1): 74–101.

[2] Engel GL. The clinical application of the biopsychosocial model. The American Journal of Psychiatry. 1980;137: 535–544. 10.1176/ajp.137.5.535 7369396

[3] Theunissen, MHC. The early detection of psychosocial problems in children aged 0 to 6 years by Dutch preventive child healthcare: professionals and their tools [dissertation]. TNO Leiden, The Netherlands; 2013.

[4] Bot M, de Leeuw den Bouter BJ, Adriaanse MC. Prevalence of psychosocial problems in Dutch children aged 8–12 years and its association with risk factors and quality of life. Epidemiol Psychiatr Sci. 2011;20: 357–65. 10.1017/s2045796011000540 22201213

[5] Reijneveld SA, Vogels AGC, Hoekstra F, Crone MR. Use of the Pediatric Symptom Checklist for the detection of psychosocial problems in preventive child healthcare. BMC Public Health. 2006;6: 1471–2458. 10.1186/1471-2458-6-197 PMC1550396 16872535

[6] Romeo R, Knapp M, Scott S. Economic cost of severe antisocial behaviour in children–And who pays it. British Journal of Psychiatry. 2006;188: 547–553. 10.1192/bjp.bp.104.007625 16738345

[7] Van Lier PAC. Preventing disruptive behavior in early elementary school children Rotterdam: Optima Grafische Communicatie; 2002.

[8] Scott S, Knapp M, Henderson J, Maughan B. Financial cost of social exclusion: follow up study of antisocial children into adulthood. British Medical Journal (Clinical Research Ed.), 2001;323(7306): 191–194. 10.1136/bmj.323.7306.191 PMC35269 11473907

[9] Marchette LK, Weisz JR. Practitioner review: Emperical evolution of youth psychotherapy toward transdiagnostic approaches. J Child Psychol Psychiatry. 2017 9:58(9): 970–984. 10.1111/jcpp.12747 28548291

[10] Caspi A, Houts RM, Belsky DW, Goldman-Mellor SJ, Harrington H, Israel S, et al The p factor: One general psychopathology factor in the structure of psychiatric disorders? Clinical Psychological Science. 2014 3; 2(2): 119–137. 10.1177/2167702613497473 25360393 PMC4209412

[11] Sauer-Zavala S, Gutner CA, Farchione TJ, Boettcher HT, Bullis JR, Barlow DH. Current definitions of “Transdiagnostic” in treatment development: A search for consensus. Behavior Therapy. 2017;48(1): 128–138. 10.1016/j.beth.2016.09.004 28077216

[12] Allen LB, White KS, Barlow DH, Shear KM, Gorman JM, Woods SW. Cognitive-behavior therapy(CBT) for panic disorder: Relationship of anxiety and depression comorbidity with treatment outcome. Journal of Psychopathology and Behavioral Assessment. 2010;32(2): 185–192. 10.1007/s10862-009-9151-3 20421906 PMC2855025

[13] DeRubeis RJ, Hollon SD, Amsterdam JD, Shelton RC, Young PR, Salomon RM, et al Cognitive therapy vs medications in the treatment of moderate to severe depression. Archives of General Psychiatry. 2005;62(4): 409–416. 10.1001/archpsyc.62.4.409 15809408

[14] McHugh RK, Murray HW, Barlow DH. Balancing fidelity and adaptation in the dissemination of empirically-supported treatments: The promise of transdiagnostic interventions. Behaviour Research and Therapy. 2009;47(11): 946–953. 10.1016/j.brat.2009.07.005 19643395 PMC2784019

[15] Brosnan R, Carr A. Adolescent conduct problems In: Carr A, editor. What works with children and adolescents: A critical review of psychological interventions with children, adolescents and their families. London: Routledge; 2000 pp. 131–154.

[16] Sukhodolsky DG, Kassinove H, Gorman BS. Cognitive-behavioral therapy for anger in children and adolescents: A meta-analysis. Aggression and Violent Behavior. 2003;9: 247–269.

[17] Greenberg MT, Domitrovich C, Bumbarger B. The prevention of mental disorders in school-aged children: Current state of the field. Prevention & Treatment. 2001;4(1): 1–62.

[18] Salmivalli C. Participant role approach to school bullying: Implications for interventions. Journal of Adolescence. 1999;22(4): 453–459. 10.1006/jado.1999.0239 10469509

[19] Vliek L, Orobio de Castro B. Stimulating positive social interaction: What can we learn from TIGER (Kanjertraining)? In: Doll B, Baker J, Pfohl B, Yoon J, editors. Handbook of Youth Prevention Science. New York: Routledge; 2010 pp. 288–308.

[20] Ryan RM, Deci EL. Self-determination theory and the facilitation of intrinsic motivation, social development, and well-being. American Psychologist. 2000;55: 68–78. 10.1037//0003-066x.55.1.68 11392867

[21] Deci EL, Ryan RM. Self-determination theory In: Van Lange PAM, Kruglanski AW, Higgins ET, editors. Handbook of theories of social psychology: Vol. 1 Sage, Thousand Oaks; 2012 pp. 416–437. 10.4135/9781446201022

[22] Topper Training Foundation. Handleiding Kanjertraining Basisonderwijs. Almere: Topper Training Foundation; 2007–2019. Dutch.

[23] Vliek L, Overbeek G, Orobio de Castro B. Improving Classroom Climate: Effectiveness of Toppertraining (Kanjertraining) in Disruptive Primary School Classes. In: Vliek L. Effects of Kanjertraining (Topper Training) on Emotional Problems, Behavioural Problems and Classroom Climate [dissertation]. Utrecht University; 2015. Available from https://www.kanjertraining.nl/wp-content/uploads/2015/08/effects_of_eanjertraining/dissertation-1.pdf

[24] Vliek L, Overbeek G, Orobio de Castro B. “I want to behave prosocially and I can choose to do so”: Effectiveness of TIGER (Kanjertraining) in 8- to 11-year-olds. European Journal of Developmental Psychology. 2014;11(1): 77–89.

[25] Goodman R. Psychometric properties of the Strengths and Difficulties Questionnaire (SDQ). Journal of the American Academy of Child and Adolescent Psychiatry, 2001;40: 1337–1345. 10.1097/00004583-200111000-00015 11699809

[26] Van Widenfelt BM, Goedhart AW, Treffers PDA, Goodman R. Dutch version of the Strengths and Difficulties Questionnaire (SDQ). European Child & Adolescent Psychiatry. 2003;12(6): 281–289. 14689260 10.1007/s00787-003-0341-3

[27] Goodman R. The extended version of the Strengths and Difficulties Questionnaire as a guide to child psychiatric caseness and consequent burden. Journal of Child Psychology and Psychiatry. 1999;40: 791–801. 10433412

[28] Van Leuven M, Van Beek Y. Children’s Depression Inventory, Nederlandse Bewerking. Internal Report. Utrecht, The Netherlands: Utrecht University; 2000. Dutch.

[29] Kovacs M. Children’s Depression Inventory, Manual. New York/Toronto: Multi-Health Systems; 1992.

[30] Kovacs MD. Children’s Depression Inventory (CDI): Technical manual. North Tonawanda, NY: Multi Health Systems Inc; 2001.

[31] Mattison RE, Handford HA, Kales HC, Goodman AL, McLaughlin RE. Four-year predictive value of the Children’s Depression Inventory. Psychological Assessment, 1990;2: 169–174.

[32] Van Beek Y. Hessen DJ, Hutteman R, Verhulp EE, van Leuven M. Age and gender differences in depression across adolescence: Real or 'bias'? Journal of Child Psychology and Psychiatry and Allied Disciplines. 2012;53(9): 973–985. 10.1111/j.1469-7610.2012.02553.x 22512614

[33] Veerman JW, Straathof MAE, Treffers PhDA, Van den Bergh BRH, Ten Brink LT. Competentiebelevingsschaal voor Kinderen: handleiding Lisse: Harcourt Assessment BV; 2004. Dutch.

[34] Harter S. Manual for the self-perception profile for children Denver, CO: University of Denver; 1988.

[35] Muris P, Meesters C, Fijen P. The Self-Perception Profile for Children: further evidence for its factor structure, reliability, and validity. Personality and individual differences. 2002; 35: 1791–1802.

[36] Vliek L, Riet B, Weide G, Overbeek G, Orobio de Castro B. Psychometric quality of the Topper questionnaire: reliability, validity and normative data. In: L Vliek. Effects of Kanjertraining (Topper Training) on Emotional Problems, Behavioural Problems and Classroom Climate [dissertation]. Utrecht University; 2015. Available from https://www.kanjertraining.nl/wp-content/uploads/2015/08/effects_of_eanjertraining/dissertation-1.pdf

[37] Olweus D. The Revised Olweus Bully/Victim Questionnaire. Mimeo. Bergen, Norway: Research Center for Health Promotion, University of Bergen; 1996.

[38] Farrington DP, Ttofi MM. School-based programs to reduce bullying and victimization. Campbell Systematic Reviews, 6; 2010. (First published: 15 December 2009; Last updated: 8 March 2010). 10.1002/cl2.1143 PMC8356322 37131921

[39] Morawska A, Mitchell AE, Burgess S, Fraser J. Effects of Triple P parenting intervention on child health outcomes for childhood asthma and eczema: Randomised controlled trial. Behavior Research and Therapy. 2016;83: 35–44. 10.1016/j.brat.2016.06.001 27295179

[40] Altman N, Krzywinski M. Points of significance: Interpreting P values. Nature Methods. 2017;14: 213–214. 10.1038/nmeth.4210 PMC5905345 29664466

[41] Wasserstein R, Lazar NA. The ASA’s statement on p-values: Context, process, and purpose. The American Statistician. 2016;70: 129–133.

