# Supplementary figures and images for: Effects of Topper Training on psychosocial problems, self-esteem, and peer victimisation in Dutch children: A randomised trial
Source: PLoS One. 2019 Nov 27;14(11):e0225504. doi: 10.1371/journal.pone.0225504 (PMC6881013; doi:10.1371/journal.pone.0225504)

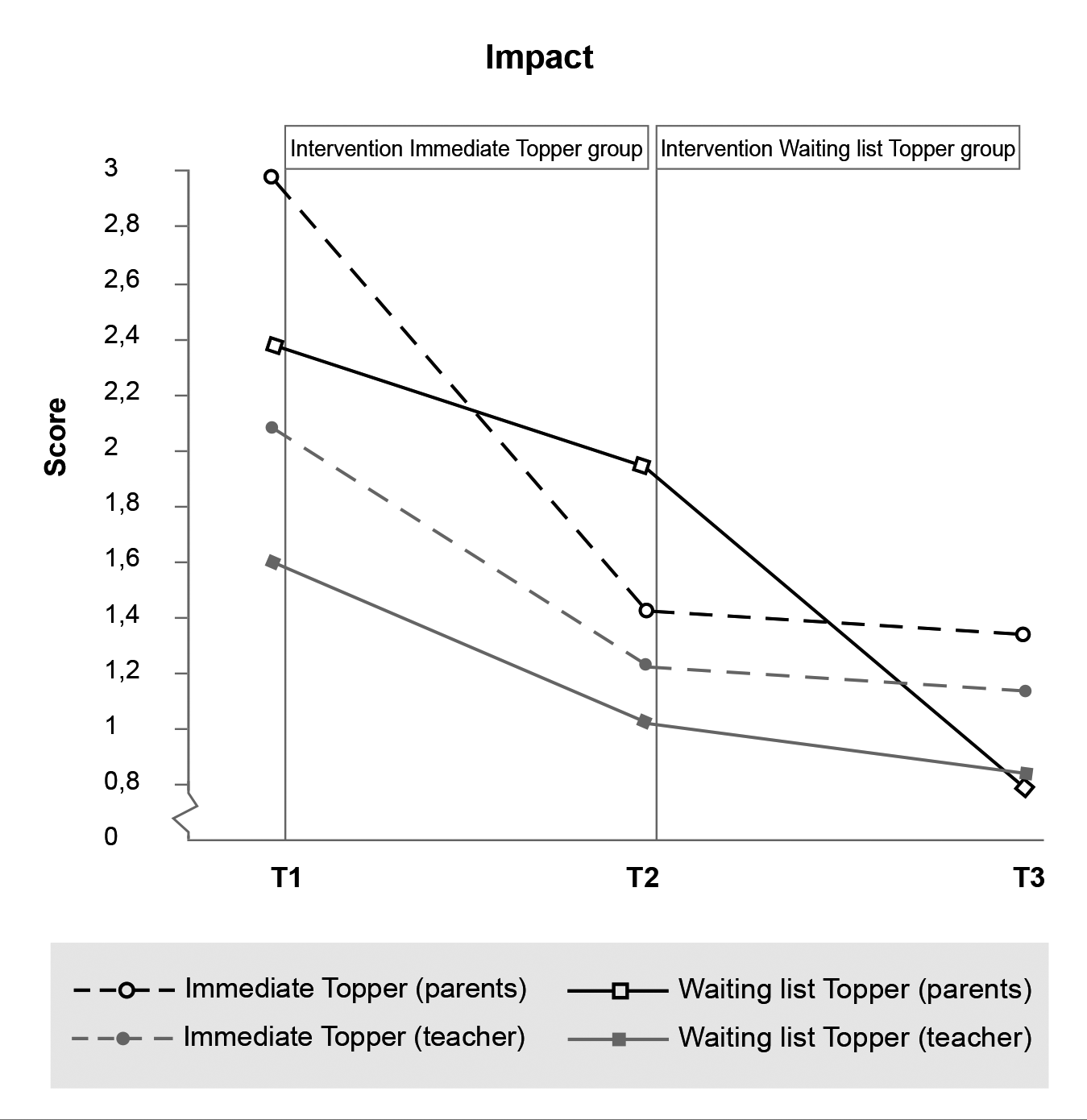

Supplement: S1 Fig — (TIF) [file pone.0225504.s001.tif]

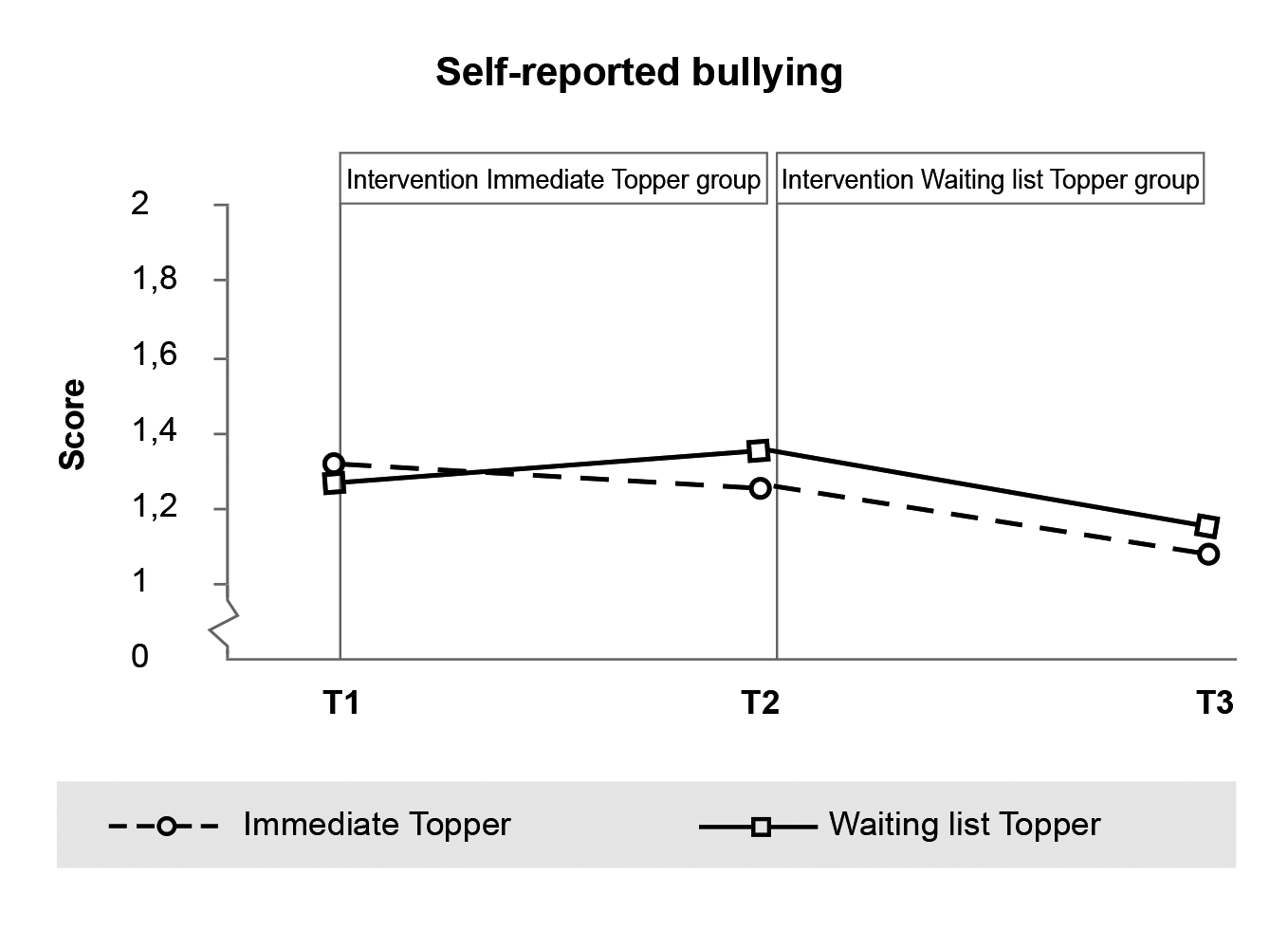

Supplement: S2 Fig — (TIF) [file pone.0225504.s002.tif]
