# Supplementary material for: Effects of Topper Training on psychosocial problems, self-esteem, and peer victimisation in Dutch children: A randomised trial
Source: PLoS One. 2019 Nov 27;14(11):e0225504. doi: 10.1371/journal.pone.0225504 (PMC6881013; doi:10.1371/journal.pone.0225504)
Supplement: S1 File — (DOC) [file pone.0225504.s003.doc]

**S1 File. Abstract in Dutch**

De meeste interventies die gericht zijn op het verbeteren van sociale interacties zijn gericht op internaliserende òf externaliserende problemen bij kinderen. Een recente review laat echter zien dat een transdiagnostische aanpak beter zou aansluiten bij de diversiteit van problemen in een groep en binnen een individu (comorbiditeit). We onderzochten het effect van een transdiagnostische interventie: Kanjertraining. Dit is een cognitieve gedragstraining die gegeven wordt in een groep met leeftijdsgenoten met diverse psychosociale problemen en hun ouders.

We voerden een gerandomiseerde trial (RCT) uit met 132 kinderen met milde tot ernstige psychosociale problemen. Hiervan kwamen 77 kinderen in de trainingsconditie en 55 in een wachtlijst conditie (50% jongens; 8 tot 11 jaar oud). We vonden significante interventie-effecten (met GLM herhaalde metingen analyses) op ouder-gerapporteerde (maar niet leerkracht-gerapporteerde) emotionele problemen (Cohen’s *d* = .70), problemen met leeftijdsgenoten (*d* = .41) en impact van de problemen op het leven (*d* = .59). Ook vonden we significante interventie-effecten op zelfgerapporteerd gepest worden (*d* = .62), zelfwaardering (*d* = .45) en gedragsproblemen volgens de leerkracht (*d* = .42). De effecten waren klinisch relevant voor ouder-gerapporteerde emotionele en gedragsproblemen, de impact van de problemen op het leven, en voor zelfwaardering. We vonden geen effecten op pesten en prosociaal gedrag; hierbij bleek sprake van een bodemeffect. Alle effecten bleven behouden een half jaar na de training. Depressie nam tijdens de training niet meer af in de trainingsgroep dan in de controlegroep. Een half jaar na de training bleek depressie wel significant te zijn afgenomen ten opzichte van vlak na de training. Concluderend: kinderen met milde tot ernstige internaliserende en/of externaliserende problemen kunnen baat hebben bij Kanjertraining.
