# Supplementary material for: Effects of Topper Training on psychosocial problems, self-esteem, and peer victimisation in Dutch children: A randomised trial
Source: PLoS One. 2019 Nov 27;14(11):e0225504. doi: 10.1371/journal.pone.0225504 (PMC6881013; doi:10.1371/journal.pone.0225504)
Supplement: S2 File — (DOC) [file pone.0225504.s004.doc]

**S2 File. Trial study protocol in Dutch**

**Onderzoek naar de effecten van de Kanjertraining op de praktijk**

**Doelgroep**: Kinderen met sociale problemen tussen 8 en 12 jaar

**Onderzoeksvraag**: Wat zijn de effecten van de Kanjertraining op depressieve gevoelens, het gedrag van kinderen (volgens ouders, leerkracht en kind) en zelfwaardering?

**Design**:

Bij iedere aanmelding worden eerst vragenlijsten opgestuurd. Daarna moet er gedobbeld worden of kinderen controle- of trainingkind zijn (zonder inzage in vragenlijsten).

Om te zorgen dat we zoveel mogelijk trainingskinderen krijgen (ethisch motief), kiezen we een ratio van 3:2. Dobbelen: 1,2,3 = training. 4,5 is wachtlijst, 6= dobbel nog een keer. Administratie dobbelt en mag niet opnieuw dobbelen, behalve bij 6.

Data van metingen en trainingen zijn te zien in tabellen hieronder.

Richtlijn: in totaal 90 kinderen: ongeveer 54 training en 36 controlekinderen

Voor de helderheid noem ik hieronder de groepen naar wanneer ze getraind worden.

Groep A: sep2010groep

Groep B: feb2011groep

Groep C: sep2011groep

**Meetinstrumenten**

**Ouders**

SDQ

**Kind**

Kanjerlijst (meet pesten en gepest worden)

CBSK (meet zelfwaardering)

CDI (meet depressie)

**Leerkracht**

SDQ

**Analyseplan**

Voer herhaalde metingen analyses uit. Een interactie-effect (groep x meting) duidt op een effect van Kanjertraining. Bekijk ook klinische relevantie van de resultaten: hoeveel kinderen die klinisch scoren op de voormeting gaan gezond weer naar huis?

Lange termijn effect is alleen te meten binnen de trainingsgroep. Voer gepaarde t-toetsen uit tussen T2 en T3 met data van de trainingsgroep.

**Procedure**

- Lilian werft kinderen via scholen, maakt posters voor op scholen, bij OK punten en huisarts in ons gebouw. Dit is in periode tussen februari en juni 2010. Begin mei moeten we genoeg kinderen hebben die zich hebben aangemeld.
- Rond 1 mei sturen we alle kinderen de lijsten toe met brief uitleg onderzoek
- Meting1: opsturen lijsten naar ouders: SDQ en het toestemmingsformulier, SDQ voor leerkracht, en voor kind de kanjerlijst.
- Na ontvangst vragenlijsten: loting september/januari training. Let op: van belang dat dit echt met dobbelsteen gebeurt: geen voorrang aan speciale gevallen.
- Eind augustus nog een keer meten want er zit anders 4 maanden tussen voormeting en begin training. Dan hebben we van alle kinderen twee voormetingen.

**Samenvattend invullen van de lijsten**

- Voormeting 1: Mei 2010:
  - Controlegroep: ze moeten wachten op een intakegesprek: In dat geval vullen ouders de SDQ en de NOSI via de post in en de kinderen de Kanjerlijst. Kinderen vullen dan op school de CBSK en de CDI in en andere lijst(en).
  - Trainingsgroep: via de post vullen ouders SDQ en NOSI in en de kinderen de Kanjerlijst. Vlak voor het intakegesprek vult het kind de CBSK en CDI in.
- Extra voormeting in augustus:
  - Trainingkinderen: voor de eerste les vullen ouders en kinderen de lijsten nog een keer in. Half uur eerder inplannen.
  - Controlekinderen: lijsten voor ouders worden weer opgestuurd. Kinderen worden op school gemeten.
- Nameting december 2010
  - Trainingskinderen direct na diploma-uitreiking
  - Controlekinderen: lijsten voor ouders worden weer opgestuurd. Kinderen worden op school gemeten.
- Nameting2 mei 2011
  - Terugkomdag voor trainingsgroep september 2010
  - Na diplomauitreiking voor trainingsgroep februari 2011
  - Voor intakegesprek voor training september 2011.
- Hoe lang gaan we door? Dit is afhankelijk van aantal kinderen die eerste keer meedoen.

Overzicht van meetmomenten en trainingen per groep. Waar een x staat, worden lijsten ingevuld.

|  | **2010** | | | | **2011** | | | | |  |
| --- | --- | --- | --- | --- | --- | --- | --- | --- | --- | --- |
| **Meting** | **Mei**  **M0510** | **Aug.**  **M0810** |  | **Dec.**  **M1210** |  | **Mei**  **M0511** | **Aug.**  **M0811** |  | **Dec.**  **M1211** | **Mei**  **M0512** |
| **Training** |  |  | **Sept-dec** |  | **Febr.-mei** |  |  | **Sept-dec** |  |  |
| sep2010groep1 en 2 | x | x | Training1 | x |  | x |  |  |  |  |
| feb2011groepA 1 en 2 | x | x | wachtlijst | x | Training2 | x |  |  | x |  |
| feb2011groepB 1 en 2 |  |  |  | x | Training2 | x | x |  | x |  |
| Sept2011groep 1 en 2 |  |  |  | x | wachtlijst | x | x | Training3 | x | x |

**Planning 2010**

| **DATUM** | **maart-mei ‘10** | **Mei 2010** | **Als lijsten binnenkomen** | **Mei-juni**  **2010** | **Mei** | **Eind augustus** | **25 aug 2010** | **Sept-dec**  **2010** | **November 2010** | **Als lijsten binnenkomen** | **december** | **dec 2010- jan. 2011** | **Begin dec 2010** |
| --- | --- | --- | --- | --- | --- | --- | --- | --- | --- | --- | --- | --- | --- |
| **WAT?**  **WIE?** | **Werving** | **Meting1** | **Toewijzing groepen, bellen** | **Intake**  **gesprekken sept. groep** | **Controlegroep kinderen meten op school** | **Controlegroep kinderen meten op school** | **Start training** | **Werving febr/sept**  **2011 groep** | **Meting opsturen** | **Toewijzing groepen, bellen** | **Controlegroep meting** | **Intake**  **gesprekken jan. groep** | **Eind training, meting2** |
| **Lilian** | Werf kinderen via scholen |  |  |  | begeleiden vragenlijsten invullen | begeleiden vragenlijsten invullen | Invullen vragenlijsten voor de training, begeleiding hierbij | Werf kinderen via scholen |  |  | Op scholen kinderen meten |  | Maak lijsten in orde, afname na diploma-uitreiking |
| **administratie** |  | Stuur lijsten naar alle ouders | Dobbelsteen selectie sept/feb-groep. Bel ouders en plan intakegesprek (Septgroep) of geef aan lijsten weer invullen in augustus en december, kinderen op school. | Tineke en Elly zijn vraagbaak bij invullen lijsten |  |  |  |  | Stuur lijsten naar ouders van jan. groep: kanjerlijst, SDQ, SDQ lk, meenemen naar intake gesprek | Dobbelsteen selectie feb/sept 2011 groep. Bel ouders en plan intakegesprek (febr.groep) of geef aan lijsten weer invullen in mei en augustus, kinderen op school. |  |  |  |
| **Trainers: Annemieke/Bas** |  |  |  | Intakegesprekken, kind vult vóór intake CBSK en CDI in. |  |  | Training geven. Pas komen nadat vragenlijsten zijn ingevuld. |  |  |  |  | Intake  gesprekken, kind vult vóór intake CBSK en CDI in |  |
| **Sept2010groep** | x | x | x | x |  |  | x |  |  |  |  |  | x |
| **Feb2011groep** | x | x | x |  | x | x |  |  | x |  |  | x |  |
| **Sept2011groep** |  |  |  |  |  |  |  | x |  |  | x |  |  |

**Planning 2011**

| **DATUM** | **februari 2011** | **Mei 2011** | **Mei 2011** | **Mei 2011** | **Mei-juni 2011** | **Eind augustus** | **Eind augustus** | **Eind december** | **Eind decemeber** | **Evt. mei 2012** |
| --- | --- | --- | --- | --- | --- | --- | --- | --- | --- | --- |
| **WAT?**  **WIE?** | **Start training feb2011groep** | **Eind training**  **Meting** | **Terugkomdag Meting** | **Meting voor sept2011 groep** | **Intake**  **gesprekken sept2011 groep** | **Invullen lijsten** | **Training sept2011 groep** | **Meting na training** | **Follow up febr2011 groep** | **Follow-upmeting** |
| **Lilian** |  | Maak lijsten in orde, aanwezig bij afname na diploma-uitreiking | Vragenlijsten laten invullen, uitleg onderzoek evt. |  |  | Kinderen en ouders vullen een half uur voor les 1 de lijsten weer in |  | Na diploma vullen ouders en kinderen de lijsten weer in. | Terugkomdag van februari 2011 groep. Ouders en kinderen vullen lijsten weer in | Terugkomdag met vragenlijsten afnemen. |
| **administratie** |  |  |  | Stuur lijsten naar ouders van sept2011. groep: kanjerlijst, SDQ, SDQ lk meenemen naar intake gesprek |  |  |  |  |  |  |
| **Annemieke/Bas** | Training geven |  | Spelletje |  | Intakegesprekken, kind vult vóór intake CBSK en CDI in |  | Training geven |  | Spelletje |  |
| **Sept2010groep** |  |  | x |  |  |  |  |  |  |  |
| **Feb2011groep** | x | x |  |  |  |  |  |  | x |  |
| **Sept2011groep** |  |  |  | x | x | x | x | x |  | x |
