# Supplementary material for: Effects of Topper Training on psychosocial problems, self-esteem, and peer victimisation in Dutch children: A randomised trial
Source: PLoS One. 2019 Nov 27;14(11):e0225504. doi: 10.1371/journal.pone.0225504 (PMC6881013; doi:10.1371/journal.pone.0225504)
Supplement: S3 File — (DOC) [file pone.0225504.s005.doc]

**S3 File. Trial study protocol in English**

Studying the effects of Topper Training in mental health care

**Target group**: Children with psychosocial problems between 8 and 12 years

**Research question:** What are the effects of Topper Training on depressive feelings, the behavior of children (according to parents, teacher and child) and self-esteem?

**Design**:

With every application, first questionnaires are filled in. Thereafter a dice is thrown whether children are control or training children (without insignts in the questionnaires).

To maximize the number of children in treatment (ethical motive), we choose a ratio of 3:2: 1,2,3 on de the dice = intervention, 4,5 is waiting list, 6 = throw again.

Data and measurement timepoints are shown in the tables below.

I call below the groups according to when they are trained.

Group A: sep2010 group

Group B: Feb2011 group

Group C: sep2011 group

**Measures**

**Parents**

SDQ

**Child**

Kanjerlijst (measures bullying and victimisation)

CBSK (measures global self-esteem)

CDI (measures depression)

**Teacher**

SDQ

**Analysis plan**

Perform repeated measurements analyzes. An interaction effect (group x measurement) indicates an effect of Topper Training. Look at the clinical relevance of the results: How many children who score clinically on the pre-test go home healthy? Long-term effect can only be measured within the training group. Perform paired t-tests between T2 and T3 with data from the training group.

**Procedure**

- Lilian recruits children through schools, makes posters for schools, at OK points and GPs in the building.
- Recruiting date: the period between February and June 2010. At the beginning of May we must have enough children who have registered.
- Around the first of May we send all children the lists with a letter explaining the study
- Measurement1: send questionnaires to parents: SDQ and the consent form, SDQ for teacher, and for child the Topper list.
- After receiving questionnaires: randomisation: September or January training. Note: it is important that this really happens with a dice: no priority for special cases.
- Measure again at the end of August because there is otherwise 4 months between pre-test and start of training. Then we have two preliminary measurements of all children.

**Summarizing**

• Pre-measurement 1: May 2010:

o Control group: they have to wait for an intake interview: in that case, parents fill in the SDQ and the NOSI by mail and the children enter the Kanjer list. Children then fill in the CBSK and the CDI at school and other list (s).

o Training group: parents fill in SDQ and NOSI via the mail and the children fill in the Kanjer list. Just before the intake interview, the child completes the CBSK and CDI.

• Extra pre-measurement in August 2010:

o Training children: for the first lesson parents and children fill in the lists one more time. Schedule half an hour earlier.

o Control children: lists for parents are sent again. Children are measured at school.

• post measurement December 2010

o Training children immediately after last lesson

o Control children: lists for parents are sent again. Children are measured at school.

• Follow-up May 2011

o Return day for training group September2010

o After graduation ceremony for February 2011 training group

o Befor intake interview for training September2011.

• How long do we continue? This depends on the number of children who participate the first time.

Overview of measurement timepoints and interventions per group. An x indicates that questionnaires are filled in.

|  | **2010** | | | | **2011** | | | | |  |
| --- | --- | --- | --- | --- | --- | --- | --- | --- | --- | --- |
| **Meting** | **May**  **M0510** | **Aug.**  **M0810** |  | **Dec.**  **M1210** |  | **May**  **M0511** | **Aug.**  **M0811** |  | **Dec.**  **M1211** | **May**  **M0512** |
| **Training** |  |  | **Sept-Dec** |  | **Febr.-May** |  |  | **Sept-Dec** |  |  |
| sep2010groep1 en 2 | x | x | Training1 | x |  | x |  |  |  |  |
| feb2011groepA 1 en 2 | x | x | Waiting list | x | Training2 | x |  |  | x |  |
| feb2011groepB 1 en 2 |  |  |  | x | Training2 | x | x |  | x |  |
| Sept2011groep 1 en 2 |  |  |  | x | Waiting list | x | x | Training3 | x | x |

Planning 2010

| **DATE** | **March-May ‘10** | **May 2010** | **When quest. return** | **May-june**  **2010** | **May** | **End of August** | **25 Aug 2010** | **Sept-Dec**  **2010** | **November 2010** | **When lists return** | **December** | **Dec 2010- Jan. 2011** | **Start Dec 2010** |
| --- | --- | --- | --- | --- | --- | --- | --- | --- | --- | --- | --- | --- | --- |
| **WHAT?**  **WHO?** | **Recruitment** | **Measurement1** | **Assigning into groups, call** | **Interviews**  **Sept. group** | **Assess control group in school** | **Assess control group in school** | **Start training** | **Recruit Febr/Sept**  **2011 group** | **Send questionnaires** | **Assigning into groups, call** | **Control group measurement** | **Interviews**  **Jan. group** | **End training, measurement2** |
| **Lilian** | Recruit children |  |  |  | Assist with questionnaires | Assist with questionnaires | Assist with questionnaires | Recruit children |  |  | Assess children in school |  | Print questionnaires prepare for last lesson |
| **Office** |  | Send questionnaires to parents | Randomize children with dice. Call parents and plan interview and questioannires. | Tineke and Elly are helpdesk |  |  |  |  | Send questionnaires to parents of Jan group: kanjerlijst, SDQ, SDQ lk. | Randomize children with dice. Call parents and plan interview and questioannires. For Feb/Sept 2011 group. |  |  |  |
| **Trainers: Annemieke/Bas** |  |  |  | Interview. Child fills in CBSK and CDI before interview. |  |  | Give training. Arrive after questioannires have been filed in. |  |  |  |  | Interview. Child fills in CBSK and CDI before interview. |  |
| **Sept2010group** | x | x | x | x |  |  | x |  |  |  |  |  | x |
| **Feb2011group** | x | x | x |  | x | x |  |  | x |  |  | x |  |
| **Sept2011group** |  |  |  |  |  |  |  | x |  |  | x |  |  |

**Planning 2011**

| **DATE** | **februari 2011** | **May 2011** | **May 2011** | **May 2011** | **May-June 2011** | **End of Augustus** | **End of Augustus** | **End of December** | **End of Decemeber** | **May 2012** |
| --- | --- | --- | --- | --- | --- | --- | --- | --- | --- | --- |
| **WHAT?**  **WHO?** | **Start training feb2011group** | **End training**  **Measurement** | **Measurement on the Return day forSept2010 group** | **First measurement for sept2011 group** | **Intake**  **sept2011 group** | **Measurement** | **Training sept2011 group** | **Measurement after training** | **Follow up febr2011 group** | **Follow-up** |
| **Lilian** |  | Prepare questionnaires, and assist | Prepare questionnaires, and assist |  |  | Children and parents fill in the quest. Half an hour before first lesson. |  | Prepare questionnaires and assist | Return day of Februari 2011 group. Prepare questionnairesand assist | Return day of September 2011 group. Prepare questionnaires and assist |
| **Office** |  |  |  | Send questionnaires to parents of sept2011 group: kanjerlijst, SDQ, SDQ lk |  |  |  |  |  |  |
| **Trainers: Annemieke/Bas** | Give training |  | Game |  | Interview. Child fills in CBSK and CDI before interview |  | Give Training |  | Game |  |
| **Sept2010group** |  |  | x |  |  |  |  |  |  |  |
| **Feb2011group** | x | x |  |  |  |  |  |  | x |  |
| **Sept2011group** |  |  |  | x | x | x | x | x |  | x |
