# Supplementary material for: Effects of Topper Training on psychosocial problems, self-esteem, and peer victimisation in Dutch children: A randomised trial
Source: PLoS One. 2019 Nov 27;14(11):e0225504. doi: 10.1371/journal.pone.0225504 (PMC6881013; doi:10.1371/journal.pone.0225504)
Supplement: S5 File — (DOC) [file pone.0225504.s007.doc]

**S5 File. Description of the intervention Topper Training**

In the first half hour, children and parents were trained together, after which they were trained in separate groups. After each meeting, parents were given background information and homework assignments to do at home. The sessions followed a detailed protocol. Each session started with a rehearsal of exercises from the previous sessions. Thereafter, the trainer introduced the theme of the session through a story. Children practiced social skills and made use of the four coloured caps (see introduction) in role-plays. They also discussed social themes and dilemmas. Every session ended with a physical trust-building exercise. The trainer

The first three sessions were directed at basic social skills: presenting oneself, eye contact, giving and receiving compliments, and expressing and interpreting emotions. In the fourth session, children practiced reacting to bullying and troublesome situations. Special attention was paid to bystander behaviour: children practiced ignoring or walking away from negative behaviour. The themes of the fifth, sixth and seventh sessions were showing interest in one another, trust, and friendship, respectively. In the seventh and eighth sessions, the children gave each other feedback: children received suggestions from their peers for behavioural change. The ninth session reminded children of the people who love them and stressed their worth to those people. “You don’t have to be loved by everyone to be worthwhile. Some people don’t like you and that’s fine.” The last session was the diploma ceremony. Parents were made aware of their role as a model for their child and practiced the same skills as the children. Moreover, a ‘Topper’ way of thinking and acting as a parent was taught wherein children’s positive intentions are affirmed and children’s sense of responsibility is stimulated by reducing psychological control over the child.
