# Supplementary material for: Effects of Topper Training on psychosocial problems, self-esteem, and peer victimisation in Dutch children: A randomised trial
Source: PLoS One. 2019 Nov 27;14(11):e0225504. doi: 10.1371/journal.pone.0225504 (PMC6881013; doi:10.1371/journal.pone.0225504)
Supplement: S1 Table — (DOC) [file pone.0225504.s008.doc]

**S1 Table. Pearson correlations of all outcome measures**

|  | 1 | 2 | 3 | 4 | 5 | 6 | 7 | 8 | 9 | 10 | 11 | 12 | 13 | 14 |
| --- | --- | --- | --- | --- | --- | --- | --- | --- | --- | --- | --- | --- | --- | --- |
| SDQ Parent Report |  |  |  |  |  |  |  |  |  |  |  |  |  |  |
| 1.Emotional Symptoms | 1 | 0,122 | ,26** | -0,135 | ,368** | ,332** | -0,138 | 0,074 | 0,008 | 0,015 | 0,175 | -0,081 | -,253** | ,211* |
| 2.Conduct Problems | 0,122 | 1 | ,29** | -,468** | ,387** | -0,045 | ,446** | ,383** | -,350** | ,247** | 0,015 | ,320** | -,248** | ,342** |
| 3.Peer Problems | ,263** | ,291** | 1 | -,173* | ,457** | ,235** | 0,156 | ,602** | -,243** | ,354** | ,247** | 0,058 | -,263** | ,281** |
| 4.Prosocial Behaviour | -0,135 | -,468** | -0,17 | 1 | -,247** | 0,03 | -,235** | -,235** | ,454** | -0,124 | -0,043 | -,240** | 0,066 | -,229** |
| 5.Impact of Problems | ,368** | ,387** | ,46** | -,247** | 1 | 0,171 | ,230** | ,285** | -,248** | ,308** | 0,14 | 0,065 | -0,122 | ,276** |
| SDQ Teacher report |  |  |  |  |  |  |  |  |  |  |  |  |  |  |
| 6.Emotional Symptoms | ,332** | -0,045 | ,24** | 0,03 | 0,171 | 1 | 0,005 | ,341** | 0,009 | ,451** | ,224* | -0,081 | -,227* | ,230** |
| 7.Conduct Problems | -0,138 | ,446** | 0,156 | -,235** | ,230** | 0,005 | 1 | ,352** | -,472** | ,504** | 0,104 | ,333** | -0,116 | 0,155 |
| 8.Peer Problems | 0,074 | ,383** | ,60** | -,235** | ,285** | ,341** | ,352** | 1 | -,427** | ,636** | ,290** | 0,043 | -,312** | ,341** |
| 9.Prosocial Behaviour | 0,008 | -,350** | -0,24 | ,454** | -,248** | 0,009 | -,472** | -,427** | 1 | -,370** | -0,051 | -,226* | 0,003 | -0,082 |
| 10.Impact of Problems | 0,015 | ,247** | ,35** | -0,124 | ,308** | ,451** | ,504** | ,636** | -,370** | 1 | 0,101 | 0,164 | -,186* | ,247** |
| Self reports |  |  |  |  |  |  |  |  |  |  |  |  |  |  |
| 11.Self-perceived victimisation | 0,175 | 0,015 | ,25** | -0,043 | 0,14 | ,224* | 0,104 | ,290** | -0,051 | 0,101 | 1 | -0,019 | -,296** | ,271** |
| 12.Self-reported bullying | -0,081 | ,320** | 0,058 | -,240** | 0,065 | -0,081 | ,333** | 0,043 | -,226* | 0,164 | -0,019 | 1 | -0,133 | ,187* |
| 13.Self-worth (SPPC) | -,253** | -,248** | -0,26 | 0,066 | -0,122 | -,227* | -0,116 | -,312** | 0,003 | -,186* | -,296** | -0,133 | 1 | -,717** |
| 14.Depression (CDI) | ,211* | ,342** | ,28** | -,229** | ,276** | ,230** | 0,155 | ,341** | -0,082 | ,247** | ,271** | ,187* | -,717** | 1 |

** p < .05, **p < .01*
